# Proteome Analysis of the Gametophytes of a Western Himalayan Fern *Diplazium maximum* Reveals Their Adaptive Responses to Changes in Their Micro-Environment

**DOI:** 10.3389/fpls.2019.01623

**Published:** 2019-12-17

**Authors:** Bhuvnesh Sareen, Pooja Thapa, Robin Joshi, Amita Bhattacharya

**Affiliations:** ^1^Division of Biotechnology, CSIR-Institute of Himalayan Bioresource Technology, Palampur, India; ^2^Department of Biotechnology, Guru Nanak Dev University, Amritsar, India

**Keywords:** differentially abundant proteins, edible fern, haploid, higher growth rate, osmotic changes, gametophytes clumps

## Abstract

Ferns have survived changing habitats and environmental extremes of different eras, wherein, the exploratory haploid gametophytes are believed to have played a major role. Therefore, the proteome of *in vitro* grown gametophytes of a temperate Himalayan fern, *Diplazium maximum* in response to 0 (G0), 1 (G1), and 3% (G3) sucrose was studied. A total of 110 differentially abundant protein spots (DAPs) were obtained. Among these, only 67 could be functionally categorized as unique proteins involved in various metabolic processes. Calcium dependent proteins, receptor like kinases, G proteins, proteins related to hormonal signaling and their interaction with other pathways, and regulatory proteins were recorded indicating the involvement of five different signaling pathways. DAPs involved in the activation of genes and transcription factors of signaling and transduction pathways, transport and ion channels, cell-wall and structural proteins, defense, chaperons, energy metabolism, protein synthesis, modification, and turnover were identified. The gametophytes responded to changes in their micro-environment. There was also significant increase in prothallus biomass and conversion of two-dimensional prothalli into three-dimensional prothallus clumps at 3% sucrose. The three-D clumps had higher photosynthetic surface area and also closer proximity for sexual reproduction and sporophyte formation. Highest accumulation of proline, enhanced scavenging of reactive oxygen species (ROS) and DAPs of mostly, abiotic stress tolerance, secondary metabolite synthesis, and detoxification at 3% sucrose indicated an adaptive response of gametophytes. Protein Protein Interaction network and Principal Component analyses, and qRT-PCR validation of genes encoding 12 proteins of various metabolic processes indicated differential adjustment of gametophytes to different levels of sucrose in the culture medium. Therefore, a hypothetical mechanism was proposed to show that even slight changes in the micro-environment of *D. maximum* gametophytes triggered multiple mechanisms of adaptation. Many DAPs identified in the study have potential use in crop improvement and metabolic engineering programs, phytoremediation and environmental protection.

## Introduction

The huge diversity of extant ferns that we see today is part of a lineage that diverged from other vascular plants in the Paleozoic era itself. A number of fern families are also known to have persisted for millions of years since their origin in the Cretaceous period but their diversification is reported to have occurred during the Cenozoic era (Testo and Sundue, 2016). Thus, ferns have battled with tremendous evolutionary pressures, changing habitats and environmental extremes of different eras. Innate abilities like functional and physiological shifts, phytochromes for reception of low light, desiccation tolerance, and flexible reproductive strategies have empowered ferns to adapt and persist under extreme selection pressures (Page, 2002; Suetsugu et al., 2005; Watkins et al., 2007). As a result, this group of plants has evolved into a unique flora with ability to adapt in novel niches (Suetsugu et al., 2005; Watkins et al., 2007; Watkins and Cardelús, 2012; Pittermann et al., 2013). Despite sharing about 90% of the genome, a major adaptive strategy in ferns is the existence of gametophytes and sporophytes as distinct and separate entities (Qui et al., 2012; Sigel et al., 2018). Each of these phases has characteristically different morphology, physiology, ecology, and ploidy. Among the two, the gametophytes are small, haploid, and stress tolerant entities in the life cycle of ferns. Gametophytes are believed to have evolved specific traits for more efficient adaptation to stressful environments than sporophytes (Watkins et al., 2007; Pittermann et al., 2013).

Germination of single-celled spores initiates the development of gametophytes, which differentiate further into distinctly shaped two-dimensional, multicellular, autotrophic structures (Racusen, 2002). The photosynthetic prothallus of the gametophytes bears a number of rhizoids for ion and water uptake (Cooke and Racusen, 1988). The rhizoids help the gametophytes adjust to the availability of nutritional resources in their surroundings (Bell, 1992; Alongi et al., 2009). Gametophytes also serve as exploratory organs (Pittermann et al., 2013). As a result, the availability of sufficient nutrition and moisture for reproduction and establishment of sporophytes in unique habitat niches are ensured. The habitat niches of ferns are strikingly different from the ones in which the angiosperms dominate (Schuettpelz and Pryer, 2009). Ample moisture, low salt and sucrose are the basic requirements for further growth and development of a gametophyte in the micro-environment of its habitat niche. Even a mild change in these factors triggers a developmental switchover to a different mode of growth, reproduction and structural organization (Mehra, 1972; Bell, 1992; Watkins et al., 2007; DeSoto et al., 2008; Alongi et al., 2009). While the growth and normal morphogenetic responses of gametophytes can change dramatically in response to limitations of water (Pajaron et al., 2015), increase in the micro-environmental concentration of sugar is known to trigger the transition of gametophytes towards apogamous phase of development (Alongi et al., 2009).

Even the gametophytes of genus, *Diplazium* have been reported to exhibit plasticity with respect to their reproduction efforts under stressful environment (Greer and McCarthy, 1999). These gametophytes also exhibit higher desiccation tolerance, frost resistance and changes in their reproductive potency under stress (Sato, 1982; Greer and McCarthy, 1999; Watkins et al., 2007). Therefore, a nutritionally rich popular edible species of the genus ‘*Diplazium*’ *i.e.*, *D. maximum* was selected for the present study. The species is a Polypodiales fern of temperate Himalayas (1,200–2,400 amsl). In a preliminary *in vitro* study involving different sucrose concentrations, the gametophytes of *D. maximum* were found to change their morphogenetic responses as well as mode of reproduction. Generally, a strong genetic machinery involving vital as well as novel genes/proteins drives such changes/deviations in a plant and helps them adapt to environmental changes. While a lot of information on the genes/proteins involved in such machinery is available for higher plants, very little is known about the ones in a fern gametophyte. Therefore, in the present study, the effect of sucrose mediated micro-environmental changes on the proteome of gametophytes of *D. maximum* was studied in order to understand their adaptative responses to changes in their micro-environment.

## Materials and Methods

### Plant Material

Sporophylls of *D. maximum* plants growing in the fernery of CSIR-Institute of Himalayan Bioresource Technology, Palampur (1,310 m amsl, 32.6°N and 78.19°E) were collected in butter-paper packets and dried in a vacuum desiccator for natural release of spores. The spores were treated with 0.01% mercuric chloride solution (w/v) containing one drop of Tween-20 for 2–3 min followed by several rinses with de-ionized water. This was done to remove all traces of toxic mercuric chloride. Finally, surface sterilized spores suspended in 1.0 ml of sterile de-ionized water were inoculated on 0.8% agar (w/v) gelled, full strength Knop’s (1865) medium (KM) at pH, 5.75 in 90 mm Petri plates. These were incubated under culture lab conditions for germination and development into gametophytes.

Observations on spore germination and further morphogenetic processes were recorded at regular 10 days interval. A stereozoom (Nikon, SMZ 1,500, DS L1-5M, Japan) was used to study the gametophytic prothalli for detailed information on microstructures and sex organ development. The gametophytes were also treated with chloral hydrate for clearing the tissue and recording the presence of sperm cells. Finally, these were stained with acetocarmine for 45 min and studied under a stereozoom.

### Incubation of Gametophyte on Medium Containing Different Concentrations of Sucrose

The chordate stage gametophytes were transferred to KM supplemented with 1, 3, and 6% sucrose respectively. Gametophytes at 1, 3 and 6% sucrose were designated as G1, G3, and G6, respectively. Gametophytes cultured on sucrose free medium served as control (G0). A minimum of ten replicates were maintained under culture lab conditions for each sucrose concentration as well as control. The culture lab conditions comprised of 25 ± 2°C and photoperiod of 16 h of cool fluorescent white light of 70 ± 5 µM m^−2^ s^−1^ and 8 h dark.

### Identification of Exact Time Point for Sucrose Mediated Morphogenetic Changes in Gametophytes

Gametophytes incubated on KM containing 0, 1, 3, and 6% sucrose (*i.e.*, G0, G1, G3, and G6) were visually observed for identification of the time point at which distinct morphological and morphogenetic changes were initiated. Based on these observations, three independent biological replicates of G0, G1, and G3 stages were harvested after 30 days. Since G6 showed vitrification and necrosis, this stage was not harvested for protein analysis.

### Protein Extraction

Various methods of protein extraction from *D. maximum* gametophytes were attempted. Finally, however, the method described by Valledor (18) was modified in the present study for extraction of proteins from G0, G1, and G3 stages. Each sample (100 mg) was first crushed in liquid nitrogen and then homogenized with PVPP (10 mg/ml) in 1.0 ml cold protein extraction buffer based on the reports of Valledor et al. (2014) and Suo et al. (2015). Finally, the buffer used in the present study comprised of 0.9 M sucrose, 10 mM EDTA, 0.4% β-ME, 0.1 M KCl, 4 mM DTT, 1 mM PMSF and 0.1 M Tris HCl, pH 8.8 by vigorous mixing at 4°C for 5 min. Thereafter, the homogenate was shaken with 600 µl Tris saturated phenol for 1 h at 4°C and then centrifuged at 21,000 g for 5 min at 4°C.

The phenolic phase obtained after centrifugation was collected in fresh tube and equal volume of washing buffer (*i.e.*, extraction buffer without PVPP) was added to it. Next, this was vortexed for 30 s and centrifuged again. The phenolic phase thus obtained was recovered and washed again. Finally, 1.0 ml of ice cold 0.1 M ammonium acetate was added to the phenol phase and proteins were allowed to precipitate overnight at −20°C. This was centrifuged again at 21,000 g for 5 min at 4°C to yield a pellet that was first rinsed three times with 1.0 ml of ice-cold methanol and then further three times with 1.0 ml acetone, each containing 0.07% DTT. The pellet was air dried and solubilized in 200 µl of urea lysis buffer containing 7 M urea, 2 M thiourea, 2% CHAPS, and 0.5% IPG-buffer 3-11 (Bio-Rad). Finally, the Bradford method was used to measure the protein concentrations against a standard curve prepared using bovine serum albumin (BSA).

### Two-DE and Gel Staining

Immobilized linear gradient gel strips (pH 3–10: 13 cm; GE Healthcare, Amersham Biosciences) were rehydrated with 200 µg of protein sample diluted with 500 µl IEF rehydration buffer containing 7 M urea, 2 M Thiourea, 2% CHAPS, 0.5% IPG buffer, 1.4 mg DTT and 0.002% bromophenol blue for 14 h at 20°C. Next, these were subjected to IEF in Ettan IPGphor-3 (GE Healthcare) system. While the current limit for electro-focusing of proteins was set at 50 µA current/strip as per the manufacturer’s protocol, the voltage and duration used were 1 h at 150 V and 4.5 h at 6,000 V. The gels strips were first equilibrated in equilibration buffer (50 mM Tris-HCl; pH 8.8), 6 mM urea, 30% glycerol (v/v), 2% SDS (w/v), and 0.002% (w/v) bromophenol blue) with 58 mM DTT for reduction and then alkylation with 2.5% IAA. The equilibrated gel strips were placed on top of 12.5% (w/v) vertical SDS-PAGE gel prepared as per the method of Laemmli (1970) and sealed with low melting agarose (0.8%, w/v) for two-dimensional electrophoresis (DE). Finally, electrophoresis was performed at 25 mA current/gel at room temperature (25 ± 2°C) using Amershan Biosciences SDS Separation system (Hoefer SE 600 Ruby, UK). Molecular mass markers of 116 kDa were co-electrophoresed as standards. Three technological replicates were run for each of G0, G1, and G3 in triplicate (biological replicates) and each gel was stained with Coomasie Brilliant Blue.

### Image Analysis and Detection of Differentially Abundant Proteins

The densitometer scanner (BioRad, USA) was used to scan each two-DE at a 600 dpi resolution. Proteins detected in the digitalized images of each of the three replicates were analyzed using the PDQuest software (Bio-Rad, USA). All errors were corrected and the reliability of matches increased through spot by spot analysis of each gel. Besides spot detection, landmarks were aligned and identified, and matched spots were quantified. The DAPs showing reproducibility were marked using a software and/or numbered manually, as necessary. The proteins persistently visible in at least three replicates were considered for analysis. A correlation coefficient value of 0.8 was used for preparing match sets of each of the replicate gels. A change of at least 1.5-fold spot intensity at *p* < 0.05 and also any increase or decrease were considered.

### In-Gel Digestion and MS Analysis

DAPs that were consistent in their intensities and also presence and absence in all three replicated gels were considered for mass spectrometry (MS) analysis. These DAPs were excised manually from the coomassie brilliant blue (CBB) stained gels and then trypsinized according to standard techniques. The gel slices were first de-stained with 100 mM NH_4_HCO_3_/50% acetonitrile (ACN). Next, these were dehydrated *in vacuo* followed by pre-incubation in 20 µl of trypsin solution (0.02 g/l Trypsin Gold-Promega) in 40 mM NH_4_HCO_3_/10% ACN for 1 h at room temperature. Next, the gel slices were completely immersed in digestion buffer (40 mM NH_4_HCO_3_/10% ACN) and incubated overnight at 37°C. A Matrix Assisted Laser Desorption Ionization/Time of Flight (MALDI TOF/TOF) system (Bruker, Ultraflextrame, Germany) was used for MS analysis. A standard tryptic BSA digest (Bruker Daltonics Inc, Germany) and mass standard starter kit (Bruker Daltonics Inc, Germany) were used to calibrate the system. Strongest filtered precursor ions with a user-defined threshold (S/N ratio 50) were selected for the MS/MS scan in positive mode as explained by Kaur et al., 2015.

### Protein Identification and Classification

After removing the contaminant peaks of trypsin and CHCA matrix cluster signals (**Supplementary Table S1**), the acquired MS and MS/MS data were uploaded on the Flex Analysis platform equipped with an in-house MASCOT server (ver 2.4). Next, these were compared against National Center for Biotechnology Information non-redundant (NCBInr) database for protein identification. Parameters used for database search comprised of precursor ion mass tolerance of 50 ppm, MS/MS fragment ion mass tolerance of 0.5 Da, maximum missed cleavages >1, peptide charge state +1, monoisotopic mass, trypsin enzyme, and also *viridiplantae* taxonomy. Oxidations of methionine and carbamidomethylation of cysteine were considered as modifications, wherever necessary. Number of matching peptides, the coverage, as well as pI and mass value nearest to the recorded one were considered for establishing the identities of DAPs.

### Principal Component Analysis and Heatmap

The PAST software (ver 2.17c) was used to perform the PCA analysis of the main groups of sucrose inducible DAPs from G0, G1, and G3. The aim was to identify the segregating groups of DAPs at different concentrations of sucrose. The MeV software (version 4.9.0) was used for construction of heatmap of differentially abundant proteins. Further, the DAPs were arranged for hierarchical clustering based on the distances or similarity between each other.

### Protein Protein Interaction

The protein–protein interactions were predicted using the web-tool STRING 11.0 (http://string-db.org). The DAPs of *D. maximum* were subjected to sequence BLASTing in The Arabidopsis Information Resource (TAIR) database (http://www.arabidopsis.org/Blast/index.jsp) for the identification of homologs in *Arabidopsis*. Next, an interaction network was created by subjecting the homologs to the molecular interaction tool of STRING 11.0.

### Total Ribonucleic Acid Extraction, Reverse Transcription, and Real-Time-PCR Analysis

The *iRIS* method of Ghawana et al., 2011 was used for the isolation of triplicate sets of total RNA from G0, G1, and G3. After quantification using a Nano Drop UV/Vis spectrophotometer (ND-1000, NanoDrop Technologies, USA), total RNA (1.0 µg) of each sample was used for the synthesis of first-strand cDNA by the Verso cDNA synthesis kit. Candidate genes and their sequences exhibiting best matches with ESTs of ferns from NCBI database (http://blast.ncbi.nlm.nih.gov/Blast/) were selected for validation. Specific primer pairs were designed using Primer 3.0 software (**Table S2**). The power SYBR Green PCR Master Mix (Applied Biosystems, USA) was used for qRT-PCR amplification in Applied Biosystems StepOne real time PCR (Thermofisher Scientific, USA) as per the StepOne Software v2.3 (Applied Biosystems, Thermofisher Scientific, USA). The qPCR program comprised of an initial denaturation step of 10 min at 95°C followed by 40 cycles of 95°C for 15 s, extension at 72°C for 30 s and 60°C for 60 s. At the end of the amplification cycle, a temperature ramp step was added for melting curve analysis, wherein, an initial temperature of 60°C and a final temperature of 95°C were used. Each qRT-PCR reaction was performed using three biological replicates, each having three technical replicates. The expression levels of each of the selected genes were normalized to the constitutive level of ELONGATION FACTOR 2. The relative gene expression levels were finally calculated by the 2^−ΔΔt^ method.

### Validation of Stress Response in Gametophytes Subjected to Differential Osmotic Potentials Created by Sucrose

Electrolyte conductivity, proline content and suppression of ROS scavenging enzyme activities are the hallmarks of stress responses in any living organism. Plants lose high levels of moisture under drought stress. As a result, there are drastic changes in fresh and dry weights. Hence, these were studied in the gametophytes subjected to different concentrations of sucrose. The assays/reactions were performed using three biological replicates (gametophytes) with each having three technical replicates.

### Diameter, Moisture Content, Fresh and Dry Weights of Gametophytes

The diameter and fresh weight (FW) of G0, G1, and G3 samples were recorded immediately after harvesting. The gametophyte samples were also dried at 60°C until constant weight and their dry weights (DW) were recorded. The moisture content was finally calculated as the difference between FW and DW divided by FW. The results are presented as mean value of three biological replicates with each having three technical replicates.

### Electrolyte Leakage

In order to find out whether increasing concentrations of sucrose imposed any stress on the gametophytes, the electrolyte conductivity (EC) of G0, G1, and G3 was determined as per the method of Lutts et al., 1996. The cyberscan series 6,000 (Eutech instrument PCD650, Singapore) electrical conductivity meter was used to measure the EC of each sample. The formula: Electrolyte leakage (EL)=(EC1/EC2)*100 was used for the calculation, where EC1 and EC2 represented the readings before and after autoclaving. The experiment was performed using three biological replicates (1.0 g gametophyte) with each having three technical replicates.

### Detection of Reactive Oxygen Species

The localization of ROS in G0, G1, and G3 was compared as per the slightly modified method of Osundeko et al. (2013). A hand cut transverse section of the gametophyte was first washed with de-ionized water and then treated with 5.0 µM 2’,7’-dichlorofluorescein diacetate (DCFH-DA) (Sigma Aldrich) for 3 h at 25°C. Thereafter, the excess dye was washed off using deionized water; and the sections visualized under a fluorescence microscope at 485 nm absorption and 530 nm excitation (Axio Imager, M1, Carl Zeiss, GmbH, Germany).

### Proline Content

The method of Bates et al., 1973 was used to measure the proline contents in 100 mg fresh tissues of G0, G1, and G3. The proline content calculated against a standard curve of proline (0–10 µg/ml) was expressed as µmol/100 mg fw. Three biological replicates (1.0 g gametophyte) with each having three technical replicates were taken.

### Reactive Oxygen Species Scavenging Enzyme Activity

The enzyme extract for various assays was prepared by homogenizing tissue samples of each of G0, G1, and G3 in 1.0 ml of homogenization buffer containing 2.0 mM EDTA, 1.0 mM DTT, 1.0 mM PMSF, and 0.5% Triton X-100 (v/v) in 50 mM sodium phosphate buffer (pH, 7.0). During homogenization, PVPP (50 mg) was added to the homogenate in a pre-chilled mortar and pestle. The homogenate was transferred to 1.5 ml Eppendorf tube and centrifuged at 13,000 rpm for 10 min at 4°C. Finally, the supernatant was separated out. This served as the enzyme extract for the below mentioned enzyme assays. Three biological replicates (1.0 g gametophyte) with each having three technical replicates were taken. Prior to this, the protein content of the enzyme extract was measured as per the method of Bradford (1976) and 0.2 mg/ml protein was used, irrespective of enzyme assays.

### Superoxide Dismutase EC 1.15.1.1

The method of Beauchamp and Fridovich (1971) was used to measure the SOD activity in 100 mg fresh tissue samples of G0, G1, and G3. The reaction mixture was prepared using 10 µl of enzyme extract. The activity was expressed as U/mg protein in a coupled system of riboflavin and light at a specified temperature.

### Ascorbate Peroxidase EC 1.11.1.11

The APX activity was measured as per the method of Nakano and Asada (1981). The reaction mixture comprised of 20 µl of enzyme extract (*i.e.*, 200 mg tissue sample in homogenizing buffer), 0.1 mM EDTA, 0.5 mM ascorbic acid, and 1.0 mM hydrogen peroxide (H_2_O_2_). Finally, 10 consecutive readings on the change in absorbance at 290 nm per min was recorded against blank *i.e.*, boiled homogenate in place of enzyme extract. The extinction coefficient (2800 M^−1^ cm^−1^) of ascorbate was calculated, and the enzyme activity was expressed as nmol AsA/mg protein/min based on ascorbate oxidation.

### Glutathione Reductase Activity EC 1.6.4.2

GR activity was measured as per the method of Carlberg and Mannervik (1985) using a reaction mixture containing 50 µl of enzyme extract (250 mg tissue sample). The decrease in absorbance at 340 nm per min was monitored for 5 min, and the GR activity calculated using the extinction coefficient of 6200 M^−1^ cm^−1^ for nicotinamide adenine dinucleotide phosphate hydrogen (NADPH). Finally, the GR specific activity was expressed as nmol UA/mg protein.

### Statistical Analysis

A minimum of ten replicates containing three gametophytes each was maintained under culture lab conditions. Three independent biological replicates for each sucrose concentration were harvested after 30 days of incubation. For proteomics, the experiment was repeated at least three times using freshly prepared protein samples. Data were statistically analyzed using Advance Linear/Non-linear models of General Linear Model (STATISTICA release 7, stats of Wipro, Bangalore, Karnataka, India). Analysis of variance (ANOVA) followed by Duncan’s multiple range tests were used to analyze the results of each experiment, which were repeated thrice. The means of each treatment and their interactions were compared at probability level, *P* ≤ 0.05.

## Results

### *In Vitro* Spore Germination and Gametophyte Development

**Figure 1:**
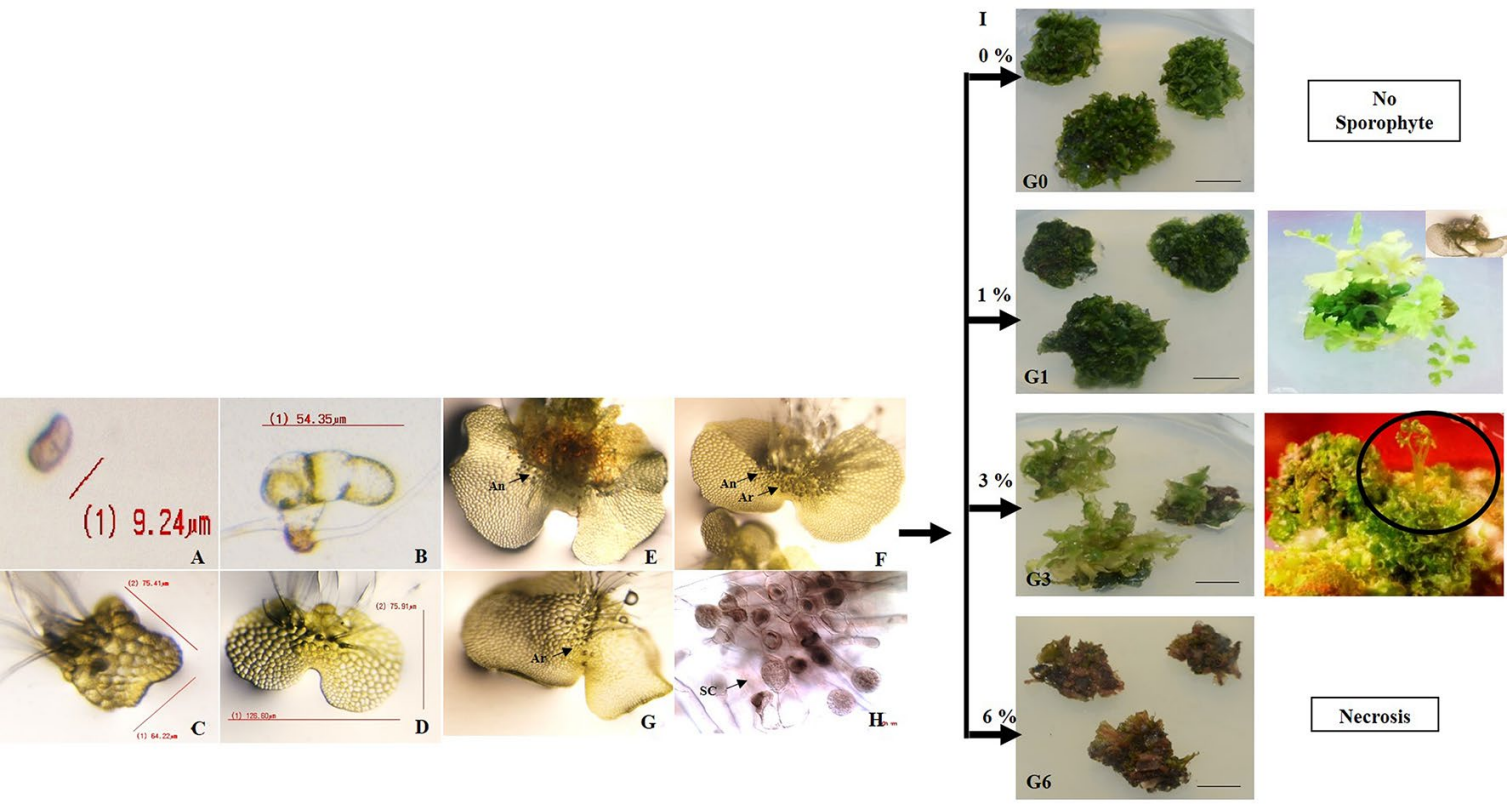
Development of gametophytes, microstructures and sporophytes in response to different sucrose concentrations. **(A)** spore **(B**–**D)** development of two-D gametophyte from microscopic spores where **(B)** protonema **(C)** spatulate stage **(D)** prothallus stage. **(E**–**H)** development of reproductive organs (antheridia and archegonia) and sperm cells, where gametophytes bearing **(E)** only antheridia (An) **(F)** only archegonia (Ar), **(G)** both An and Ar, **(H)** sperm cells (SC) within antheridium. **(I)** development of gametophytes after 30 days of incubation and sporophytes on 0.8% agar gelled Knop’s medium supplemented with 0, 1, 3, and 6% sucrose.

Asynchronous germination of spores (or emergence of the first prothallic cell) was recorded after 21–25 days of inoculation on 0.8% agar solidified Knop’s medium. The germinated spores developed into a 2–3 celled filamentous entity. While rhizoids-bearing protonema developed after 30–32 days, formation of spatulate stage was recorded after 37–40 days. The spatulate gametophytes developed further into thin, light green coloured, fully expanded chordate or the heart shaped prothalli after 48–50 days. Each of these chordate prothalli bore apical notches and numerous rhizoids at posterior side (**Figures 1A–D**). The average size of 15 randomly sampled gametophytes was 6.416 ± 0.28 mm.

The sex organs developed on the gametophytes after 50–60 days. Three different types of gametophytes were observed namely, ones (i) bearing only antheridia (ii) bearing only archegonia (iii) bearing both antheridia and archegonia (**Figures 1E–G**). The antheridia developed after 50 days of spore germination, whereas, the archegonia developed 60 days after spore germination. Detailed observations revealed a scattered localization of antheridia over the middle and basal part of the prothallus, whereas, the archegonia were present below the apical notch. The archegonia were neck shaped structures but the antheridia were spherical in shape. The presence of sperm cells was also evident within the antheridia in gametophytes cleared with chloral hydrate and stained with acetocarmine (**Figure 1H**).

### Morphogenesis of Gametophytes in Sucrose Supplemented Medium

The presence and the concentration of sucrose had a pronounced effect on the morphology and morphogenetic responses of the gametophytes during their developmental transition into sporophytes. Irrespective of sucrose concentration (1 or 3%), the gametophytes multiplied into clumps after 30 days of incubation on KM. However, the ones on 1% sucrose were loose, and individuals could be easily separated from each other (G1). The gametophytic clumps on 3% sucrose (G3) were compact, and individuals were un-separable (**Figure 1I**). While the diameter of the gametophyte clumps was 8.96 mm after 30 days of incubation on sucrose free KM, the diameter reduced slightly to 8.34 and 5.5 mm at 1 and 3% sucrose, respectively. Moreover, after 70 days on 1% sucrose, an average of 7 healthy sporophytes developed directly from each gametophyte clump. Profuse rooting was also initiated in each of these gametophytes after further 45 days (*i.e*., a total of 115 days after inoculation). In contrast, only lanky and spindly sporophytes developed from within each gametophyte clump after 165 days on 3% sucrose. An average of 15 sporophytes per gametophyte clump was recorded at this concentration. Only vitrification followed by necrosis of gametophytes was recorded within 10 days of incubation on KM containing 6% sucrose. Gametophytes at 6% (G6) were therefore, not taken for further experiments.

### Identification of Sucrose Inducible Differentially Abundant Proteins

**Figure 2:**
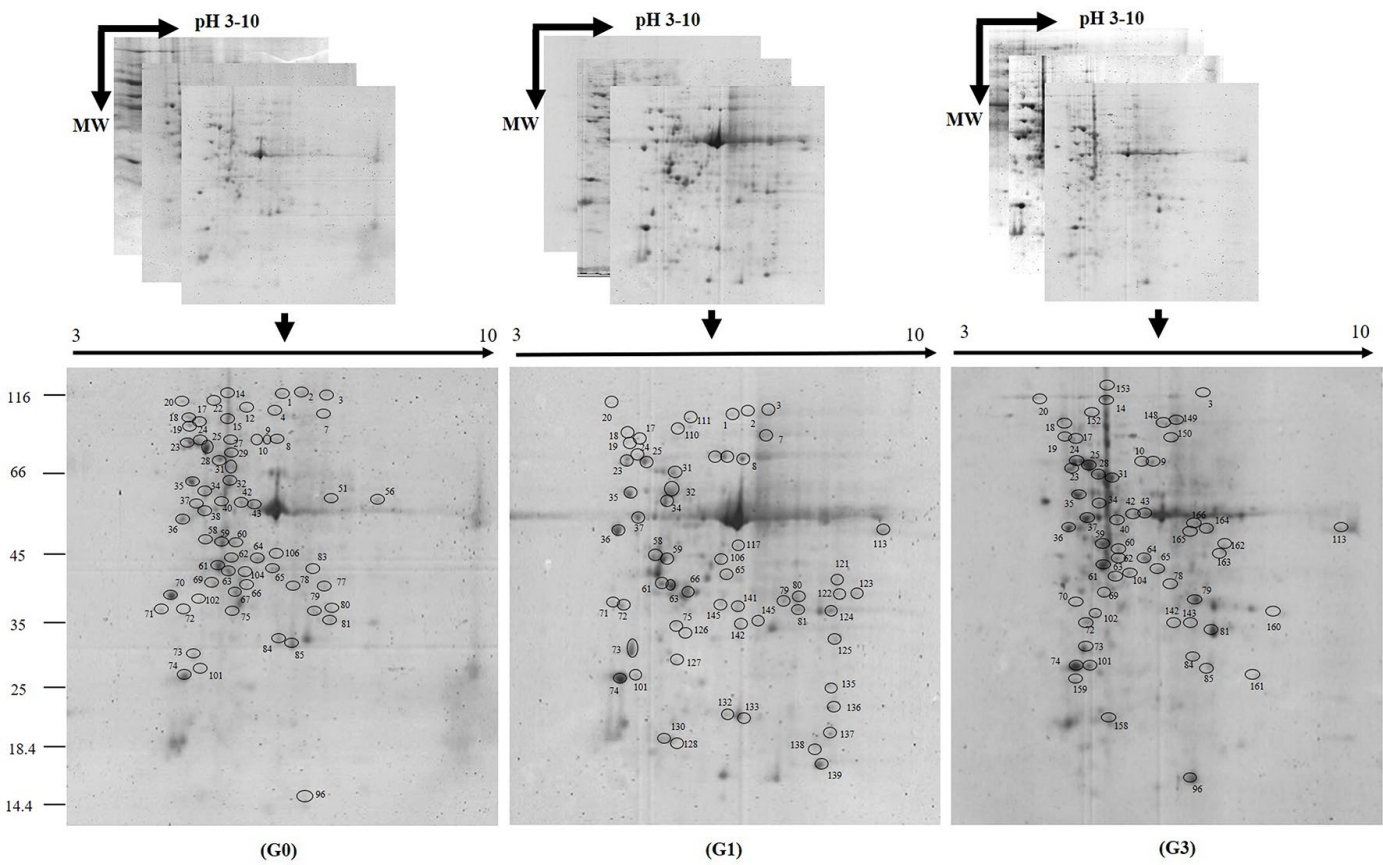
Two-dimensional gel images of proteins expressed in *Diplazium maximum* gametophytes. Three representative replicates of 2D gels of gametophytes incubated for 30 days on Knop’s medium containing 0 (G0), 1 (G1), and 3% (G3) sucrose, respectively. Proteins were separated on pH 3–10 immobilized gel strips (13 cm) and then subjected to SDS PAGE. Differentially abundant protein spots (DAPs) of G0, G1, and G3 on gels stained with Coomassie Brilliant Blue G-250 (the spots are encircled and numbered manually). A total of 110 DAPs were identified by MALDI-TOF MS/MS search.

Irrespective of sucrose treatment, a large number of protein spots were concentrated between pH 5.0 and 9.0. Their molecular weights ranged between 25 and 100 kDa. A few number of low molecular weight (18–25 kDa) spots were also present. The isoelectric points of acidic and basic proteins were abundant between pH 4.0 and 8.0. In each of the gels, more than 250 CBB stained spots were recorded, and 147 were DAP spots. Out of these 147 DAPs, a total of 110 DAPs could be identified after in-gel digestion and MALDI-TOF MS/MS search (**Figure 2**; **Figure S1**).

**Figure 3:**
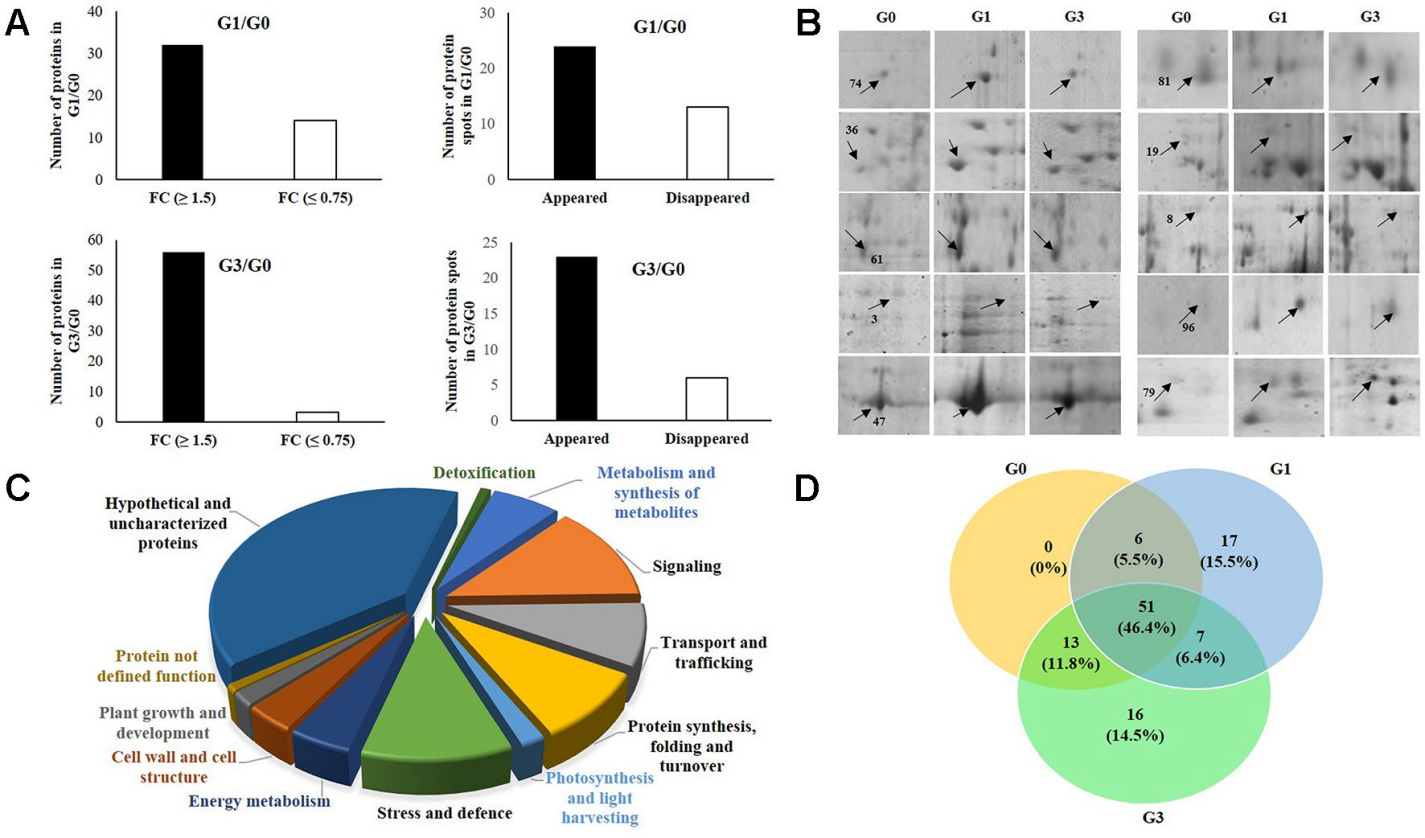
Functional categories of differentially abundant proteins of *Diplazium maximum* gametophytes after 30 days of incubation on Knop’s medium containing 0, 1, and 3% sucrose. **(A)** Status of 110 protein spots in G1 and G3 as compared to G0 where 32 in G1 and 56 in G3 exhibited a fold change (FC) of ≥1.5, whereas, 14 in G1 and 3 in G3 exhibited a fold change (FC) of ≤0.75. In addition, 24 in G1 and 23 in G3 appeared while 13 in G1 and 6 in G3 were disappeared. **(B)** Magnified view of 10 DAPs in G0, G1, and G3; The protein spots are indicated by arrows with numbers. **(C)** Pie chart showing classification of DAPs into 12 categories and their percentage composition. **(D)** Venn diagram depicting the relationship between 110 DAPs of G0, G1, and G3.

Among the total 110 proteins identified, 43 were originally annotated as unknown, hypothetical, predicted or uncharacterized. The remaining 67 were however, functionally categorized unique proteins (**Table 1**). The number of peptides, sequences, and their spectra are presented in **Table S3**. As compared to G0, 46 and 59 DAPs were recorded in G1 and G3 respectively. Furthermore, 24 and 23 spots appeared, whereas, 13 and 6 disappeared in G1 and G3, respectively (**Figure 3A**; **Table S4**). The magnified view of 10 DAPs is marked and presented in **Figure 3B**.

**Table 1:** Differentially abundant proteins of *Diplazium maximum* gametophytes in response to differential osmotic potentials created by sucrose.

Spot No.	Protein name	Accession number	Species	Thr. MM (kDa)/pI	Exp. MM (kDa)/pI	Score	Coverage (%)	Peptides (number)	Fold change G1/G0	Fold change G3/G0
**Signaling (14)**										
18	Receptor-like protein kinase FERONIA	XP_010032628.1	*Eucalyptus grandis*	98.095/5.99	98.5/5.12	69	10	10	0.68	1.55
29	NEDD8-activating enzyme E1 regulatory subunit AXR1-like	XP_020577625.1	*Phalaenopsis equestris*	59.101/5.16	77.4/5.81	65	12	11	1.52	0.86
31	CBL-interacting serine/threonine-protein kinase 13-like	XP_018481299.1	*Raphanus sativus*	56.893/8.37	68.5/5.72	67	29	15	1.57	0.86
72	VQ motif-containing protein 1	XP_021832776.1	*Prunus avium*	14.115/7.66	38.5/5.21	58	40	6	3.68	11.0
73	Calcium-dependent protein kinase 28-like	XP_022002049.1	*Helianthus annuus*	60.246/8.30	31.5/5.21	57	17	10	4.18	3.26
75	Auxin-induced protein	XP_011398343.1	*Auxenochlorella protothecoides*	32.761/5.35	35.9/5.81	65	23	7	2.89	1.56
96	Pentatricopeptide repeat-containing protein At1g12300, mitochondrial	XP_013605059.1	*Brassica oleracea* var. *oleracea*	70.265/8.02	15.5/7.14	78	20	14	3.12	3.69
101	Probable protein phosphatase 2C 21	XP_015689016.1	*Oryza brachyantha*	40.147/5.42	28.5/5.39	56	8	6	DS	4.20
109	SRSF protein kinase 1	XP_006340862.1	*Solanum tuberosum*	50.417/8.78	53.1/6.0	59	11	7	2.64	2.50
123	Gibberellin 2-beta-dioxygenase 1-like isoform X1	XP_019449246.1	*Lupinus angustifolius*	38.515/6.34	38.1/8.0	67	35	9	APP	AB
136	Shaggy-related protein kinase kappa isoform X3	XP_022854244.1	*Olea europaea* var. *sylvestris*	48.022/8.39	22.2/7.8	70	27	14	APP	AB
137	CRIB domain-containing protein RIC11-like	XP_013594423.1	*Brassica oleracea* var. *oleracea*	18.042/9.82	20.6/7.83	58	40	5	APP	AB
141	Ras-related protein RABB1c-like	XP_022926861.1	*Cucurbita moschata*	23.473/6.52	36.5/6.6	66	50	10	APP	AB
165	Probable calcium-binding protein CML45	XP_019195283.1	*Ipomoea nil*	21.177/4.56	47.2/7.16	66	29	7	AB	APP
**Stress and defense (12)**										
25	Heat shock cognate 70 kDa protein 2 isoform X1	XP_021659116.1	*Hevea brasiliensis*	71.451/5.14	79.8/5.35	134	39	26	0.42	1.06
40	23 9 kda Heat-shock protein	CAI96506.1	*Triticum dicoccon*	23.909/5.06	59.5/5.67	75	31	6	1.37	1.70
69	Patatin/Phospholipase A2-related protein	OMO74899.1	*Corchorus olitorius*	41.409/6.17	43.1/5.56	62	12	7	2.38	4.93
74	17.6 kDa class I heat shock protein 3-like	XP_004147911.1	*Cucumis sativus*	15.895/6.77	26.8/5.11	58	31	6	0.81	3.28
79	Zinc finger, CCCH-type	KVI04283.1	*Cynara cardunculus var. scolymus*	44.275/7.53	40.3/7.19	57	10	7	2.04	1.60
102	Glutathione S-transferase T3-like	XP_013721574.1	*Brassica napus*	28.475/9.07	39.1/5.62	61	25	10	DS	1.60
106	Glycine-rich RNA-binding protein RZ1A	XP_017244919.1	*Daucus carota subsp. sativus*	18.450/10.27	49.8/6.51	57	21	6	1.79	2.83
113	Protein RESTRICTED TEV MOVEMENT 2	XP_010932647.1	*Elaeis guineensis*	30.565/6.30	52.5/8.39	64	19	8	APP	APP
143	Peroxidase 27-like	XP_020256486.1	*Asparagus officinalis*	36.138/9.28	35.6/6.81	58	32	9	APP	APP
152	Phosphopantetheine adenylyltransferase isoform X1	XP_020520500.1	*Amborella trichopoda*	20.647/6.92	115.9/5.6	57	27	7	AB	APP
155	23.5 kDa heat-shock protein	CAM96555.1	*Triticum turgidum subsp. durum*	23.548/5.38	11.0/5.6	68	26	7	AB	APP
166	AP2/ERF domain-containing transcription factor, partial	APQ47426.1	*Vernicia fordii*	30.265/4.76	48.9/7.12	59	23	7	AB	APP
**Transport and trafficking (9)**										
11	Exocyst complex component EXO70A1-like	XP_013633432.1	*Brassica oleracea var. oleracea*	15.481/8.76	89.5/6.18	64	35	6	0.80	1.94
19	ABC transporter F family member 4-like isoform X1	XP_019196546.1	*Ipomoea nil*	75.698/5.17	97.5/5.08	64	18	12	0.60	3.17
59	Protein slowmo homolog	XP_002984487.1	*Selaginella moellendorffii*	21.562/8.44	51.9/5.62	64	44	11	1.15	1.58
71	Calmodulin-binding family protein	PON71702.1	*Parasponia andersonii*	58.651/9.46	38.1/4.79	66	18	11	7.15	DS
124	AP-4 complex subunit epsilon	XP_023880125.1	*Quercus suber*	108.236/5.68	36.6/7.81	63	19	16	APP	AB
126	Exocyst complex component SEC6 isoform X8	XP_024038766.1	*Citrus clementina*	72.762/5.08	33.0/5.87	60	11	11	APP	APP
130	Ras-related protein Rab-2-B	XP_003575246.1	*Brachypodium distachyon*	23.238/6.90	18.9/5.42	65	43	6	APP	APP
138	Putative protein transport Sec1b	EMS48081.1	*Triticum urartu*	84.689/8.85	18.2/7.6	63	13	9	APP	AB
145	Mechanosensitive ion channel protein 6-like	XP_022135714.1	*Momordica charantia*	109.019/8.40	37.2/6.4	61	26	24	APP	AB
**Protein synthesis, folding, and turnover (10)**										
3	F-box protein At2g39490	XP_024625966.1	*Medicago truncatula*	47.075/7.90	112.7/7.21	65	26	12	0.15	0.10
4	Maturase K, partial	ABD64648.1	*Cissampelos pareira*	58.208/9.71	100.5/6.59	55	12	9	0.69	1.59
7	Ubiquitin-like	XP_015055115.1	*Solanum pennellii*	13.299/5.85	98.5/6.91	56	33	4	1.57	1.62
8	E3 ubiquitin-protein ligase rnf12-like protein	PNY08809.1	*Trifolium pratense*	21.084/7.66	86.5/6.62	60	16	6	4.51	2.29
20	Maturase K (chloroplast)	AGC70797.1	*Mirabilis jalapa*	60.229/9.73	106.5/4.9	55	9	8	0.25	1.63
81	Ribosomal protein S14 (chloroplast)	YP_009424121.1	*Schizaea pectinata*	11.786/11.52	35.4/7.41	55	35	6	1.72	2.24
83	DNA gyrase subunit B, chloroplastic/mitochondrial-like	XP_021665185.1	*Hevea brasiliensis*	82.756/7.59	42.6/7.08	58	13	8	3.70	4.71
117	Protein RMD5 homolog A	XP_015644468.1	*Oryza sativa Japonica Group*	43.845/5.97	52.9/6.63	68	15	11	APP	AB
127	Protein RNA-directed DNA methylation 3-like	XP_017233924.1	*Daucus carota subsp. sativus*	133.816/9.57	29.1/5.79	66	14	19	APP	AB
159	E3 ubiquitin protein ligase DRIP2	XP_024462584.1	*Populus trichocarpa*	45.883/9.17	23.0/5.32	58	17	9	AB	APP
**Metabolism and synthesis of metabolites (7)**										
22	Aldehyde dehydrogenase family 2 member C4	OAY63375.1	*Ananas comosus*	52.764/5.58	112.5/5.61	61	27	18	DS	4.71
61	Molybdopterin synthase catalytic subunit	XP_010532728.1	*Tarenaya hassleriana*	21.623/6.14	42.7/5.72	78	25	9	0.47	2.79
66	Bifunctional riboflavin kinase/FMN phosphatase isoform X1	XP_024632479.1	*Medicago truncatula*	41.314/5.96	44.1/5.96	64	27	9	2.36	10.09
78	Putative pectate lyase 2	XP_017636994.1	*Gossypium arboreum*	39.646/9.31	40.8/6.82	58	20	8	DS	1.95
142	Flavanone 3-hydroxylase	AAT68774.1	*Camellia sinensis*	41.780/5.61	35.1/6.64	81	31	11	APP	APP
150	Galacturonate, partial	ADK27707.1	*Rosa roxburghii*	27.211/6.43	104.0/6.8	62	51	10	AB	APP
164	4-hydroxy-3-methylbut-2-enyl diphosphate reductase-like isoform X1	XP_022002381.1	*Helianthus annuus*	33.664/5.15	48.1/7.39	63	27	10	AB	APP
**Energy metabolism (5)**										
12	ATPase family AAA domain-containing protein 3C	XP_020887398.1	*Arabidopsis lyrata subsp. lyrata*	71.082/9.06	107.1/6.0	64	15	13	1.58	DS
32	ATP synthase CF1 alpha subunit (chloroplast)	YP_009425181.1	*Asplenium pekinense*	54.812/5.36	66.0/5.79	140	33	20	1.20	0.45
36	ATP synthase subunit beta, mitochondrial	XP_004135069.1	*Cucumis sativus*	59.885/5.90	52.9/5.05	68	33	13	1.59	1.69
125	ATP synthase beta subunit, partial (chloroplast)	ADQ64354.1	*Elephantomene eburnea*	38.264/5.26	33.7/7.9	68	52	14	APP	AB
158	Cytochrome P450	KVH06262.1	*Cynara cardunculus* var. *scolymus*	40.040/8.61	22.3/7.59	64	32	8	AB	APP
**Cell wall and cell structure (4)**										
14	Katanin p80 WD40 repeat-containing subunit B1 homolog isoform X2	XP_024046352.1	*Citrus clementina*	89.201/7.80	116.9/5.67	66	21	19	DS	2.45
17	Probable glycosyltransferase At5g03795	XP_010519887.1	*Tarenaya hassleriana*	65.901/9.43	100.5/5.24	54	8	11	DS	2.29
27	Probable microtubule-binding protein TANGLED	XP_012438336.1	*Gossypium raimondii*	11.023/9.89	89.5/5.63	68	50	9	0.74	3.69
58	Actin-related protein 2/3 complex subunit 3	XP_003571891.1	*Brachypodium distachyon*	19.685/8.87	51.9/5.52	62	41	8	0.90	1.59
	**Photosynthesis and light harvesting (2)**									
42	Ribulose-1,5-bisphosphate carboxylase/oxygenase large subunit, partial (chloroplast)	ABG48669.1	*Microgramma squamulosa*	49.400/6.54	56.7/5.97	150	50	30	0.30	1.61
62	Ribulose bisphosphate carboxylase/oxygenase activase 1, chloroplastic isoform X4	XP_024521402.1	*Selaginella moellendorffii*	73.800/5.20	45.0/5.89	68	21	16	0.81	1.51
**Plant growth and development (2)**										
65	Glycine-rich protein DOT1-like	XP_008653903.1	*Zea mays*	12.524/3.91	42.1/6.63	61	30	7	2.00	1.54
160	EPIDERMAL PATTERNING FACTOR-like protein 2	XP_006346821.1	*Solanum tuberosum*	12.365/8.83	34.9/8.31	69	33	7	AB	APP
	**Detoxification (1)**									
63	Rhodanese-like domain-containing protein 19, mitochondrial isoform X2	XP_009382034.1	*Musa acuminata subsp. malaccensis*	13.488/5.39	42.8/5.8	66	33	5	1.57	1.62
**Protein with no defined function (1)**										
148	Putative cysteine-rich repeat secretory protein 21	XP_010466466.1	*Camelina sativa*	30.104/9.25	106.0/6.58	59	48	9	AB	APP
**Hypothetical and uncharacterized proteins (43)**										
1	Predicted protein	XP_001421870.1	*Ostreococcus lucimarinus*	65.129/9.11	116.4/6.1	70	13	11	DS	4.71
2	Hypothetical protein OsI_25859	EEC81964.1	*Oryza sativa Indica*	10.962/9.80	116.6/6.9	72	63	9	3.23	DS
9	Uncharacterized protein	XP_010108696.1	*Morus notabilis*	61.171/6.90	87.0/6.25	60	8	9	2.29	10.47
10	Hypothetical protein CRG98_037683	PKI41933.1	*Punica granatum*	18.812/6.43	87.0/6.25	62	29	5	0.45	4.24
15	Hypothetical protein CRG98_010592	PKI69014.1	*Punica granatum*	19.328/4.96	105.9/5.68	60	14	5	DS	3.18
23	Uncharacterized protein LOC109820622	XP_020242387.1	*Asparagus officinalis*	16.840/5.02	85.5/5.18	66	40	9	2.81	3.28
24	Hypothetical protein JCGZ_04055	KDP38702.1	*Jatropha curcas*	69.207/5.21	86.5/5.25	68	27	15	1.04	1.50
28	Conserved hypothetical protein	EEF24426.1	*Ricinus communis*	11.910/11.67	76.9/5.61	64	31	6	1.78	1.69
34	Hypothetical protein KK1_041423	KYP37388.1	*Cajanus cajan*	6.596/9.58	63.5/5.4	63	98	5	4.33	10.57
35	Hypothetical protein AXG93_702s1060	OAE29553.1	*Marchantia polymorpha subsp. Ruderalis*	76.164/9.43	64.9/5.2	70	15	11	DS	0.90
37	Hypothetical protein EUGRSUZ_F03718	KCW70512.1	*Eucalyptus grandis*	44.933/5.14	60.5/5.38	86	28	12	2.52	1.85
38	Predicted protein, partial	BAJ94776.1	*Hordeum vulgare subsp. vulgare*	20.656/9.56	52.7/5.39	63	31	7	DS	1.90
43	Hypothetical protein COLO4_01805, partial	OMP13375.1	*Corchorus olitorius*	13.841/6.66	60.9/6.14	57	53	7	0.91	3.08
51	Hypothetical protein F511_11743	KZV38645.1	*Dorcoceras hygrometricum*	19.403/9.71	62.9/7.38	66	34	9	0.82	1.79
56	Hypothetical protein GQ55_7G208200	PUZ47967.1	*Panicum hallii* var. *hallii*	78.663/8.53	63.1/8.05	58	19	12	DS	2.77
60	Hypothetical protein LR48_Vigan03g182600	KOM38444.1	*Vigna angularis*	21.274/4.97	59.6/5.83	63	19	6	1.12	2.29
64	Uncharacterized protein LOC21398768	XP_010108696.1	*Morus notabilis*	61.171/6.90	39.6/6.25	61	10	9	1.80	1.86
67	Hypothetical protein GOBAR_AA34479	PPR86212.1	*Gossypium barbadense*	17.566/7.98	36.1/5.98	67	21	5	0.14	2.00
70	Uncharacterized protein LOC111829959	XP_023636043.1	*Capsella rubella*	25.349/8.87	39.8/4.91	60	12	5	0.42	2.75
77	Hypothetical protein PHYPA_023536, partial	PNR33720.1	*Physcomitrella patens*	5.658/8.59	40.2/7.23	64	80	5	DS	3.01
80	Hypothetical protein MNEG_5839	XP_013901142.1	*Monoraphidium neglectum*	56.896/10.09	36.2/7.41	59	18	10	1.59	1.53
84	Unnamed protein product	CDP20718.1	*Coffea canephora*	49.590/9.24	35.2/6.62	64	17	7	1.64	DS
85	Hypothetical protein VITISV_025075	CAN65323.1	*Vitis vinifera*	9.459/9.70	32.2/6.81	55	35	6	0.48	0.67
97	Hypothetical protein TRIUR3_34013	EMS54852.1	*Triticum urartu*	20.268/5.53	54.8/9.63	60	28	6	DS	0.79
98	Hypothetical protein AT4G16060	NP_193341.3	*Arabidopsis thaliana*	33.047/6.92	31.2/9.62	55	22	7	0.25	DS
103	Uncharacterized protein A4U43_C01F20550	ONK80680.1	*Asparagus officinalis*	34.568/8.95	34.1/5.65	61	23	10	5.56	2.15
104	Uncharacterized protein LOC105781050 isoform X2	XP_012461079.1	*Gossypium raimondii*	43.714/8.23	40.2/6.21	56	11	8	1.80	DS
108	Uncharacterized protein LOC108225632	XP_017256050.1	*Daucus carota subsp. sativus*	137.242/8.97	54.9/7.18	56	5	9	2.60	1.85
110	Uncharacterized protein LOC109794716	XP_020209750.1	*Cajanus cajan*	18.666/9.99	108.0/5.9	66	35	8	APP	AB
111	Hypothetical protein TRIUR3_04918	EMS66681.1	*Triticum urartu*	47.295/9.70	106.6/5.82	69	16	10	APP	AB
121	Hypothetical protein VOLCADRAFT_120394	XP_002946989.1	*Volvox carteri f. nagariensis*	35.946/6.38	45.9/7.82	66	47	15	APP	AB
122	Predicted protein	XP_002502890.1	*Micromonas commoda*	34.859/8.76	43.3/7.82	66	27	9	APP	AB
128	Uncharacterized protein LOC105782132 isoform X1	XP_012462101.1	*Gossypium raimondii*	27.755/5.23	18.7/5.65	63	36	7	APP	APP
132	Uncharacterized protein LOC109163844	XP_019168105.1	*Ipomoea nil*	36.147/6.00	21.9/6.43	63	34	10	APP	APP
133	Hypothetical protein GLYMA_01G118500	KRH75934.1	*Glycine max*	8.660/4.72	21.6/6.8	64	80	6	APP	AB
135	Hypothetical protein PRUPE_I003600	ONH89503.1	*Prunus persica*	59.676/7.66	25.8/7.8	65	22	9	APP	AB
139	Uncharacterized protein LOC18422773 isoform X1	XP_020526468.1	*Amborella trichopoda*	71.663/8.48	17.2/7.60	61	26	15	APP	AB
149	Uncharacterized protein LOC111912866	XP_023764365.1	*Lactuca sativa*	17.476/4.98	106.9/6.7	70	45	10	AB	APP
153	Uncharacterized protein LOC111315162	XP_022772481.1	*Durio zibethinus*	263.669/4.80	118.0/5.67	67	12	25	AB	APP
161	Hypothetical protein GLYMA_01G118500	KRH75934.1	*Glycine max*	8.660/4.72	25.3/8.0	65	79	5	AB	APP
162	Hypothetical protein PHAVU_003G146800g	XP_007154775.1	*Phaseolus vulgaris*	14.690/9.50	45.1/7.57	61	33	5	AB	APP
163	Hypothetical protein GLYMA_01G118500	KRH75934.1	*Glycine max*	8.660/4.72	43.7/7.57	75	79	5	AB	APP
168	Hypothetical protein OsJ_21377	EEE65732.1	*Oryza sativa Japonica Group*	43.938/6.32	35.3/7.62	58	8	6	AB	APP

Where, APP, Appeared in comparison to G0; DS, Disappeared in comparison to G0; AB, Absent in both the stages.

### Functional Classification of Differentially Abundant Proteins and Function-Based Categories

When the above identified 110 DAPs in G0, G1, and G3 were characterized, only 67 (60.9%) could be categorized functionally into 12 groups based on gene ontology, blast alignments, and literature review. The functions of the remaining 39.09% proteins were unknown. Among the ones whose functions could be assigned, there was a dominance of DAPs of signaling (12.72%) followed by those of stress and defense (10.91%). The other functional categories included DAPs of nucleotide processing, folding and turnover (9.09%), transport and trafficking (8.18%), metabolism and synthesis of structural compounds (6.36%), carbohydrate and energy metabolism (4.55%), cell wall and cell structure (3.64%), photosynthesis and light harvesting (1.82%), plant growth and development (1.82%), proteins involved in detoxification (0.09%), and proteins without specified function (0.09%) (**Figure 3C**).

### Relationship Between the Functionally Annotated Differentially Abundant Proteins of Gametophytes at 1 and 3% Sucrose

A Venn’s diagram illustrating the relationships between the DAPs of G0 *vs* G1 and G0 *vs* G3 revealed no exclusive DAPs in G0 (**Figure 3D**). However, 17 and 16 DAPs were exclusive to G1 and G3, respectively. The function(s) of a major portion of these DAPs (*i.e.*, 41.18 and 37.5%) in each of G1 and G3 was actually unknown. The next dominant but exclusive category in G1 was signaling (23.53%) followed by transport (17.65%), protein synthesis, folding and turnover (11.76%). Only 5.88% of DAPs belonged to the category of carbohydrate and energy. In case of G3, the most dominant category after ‘DAPs with unknown function’ was stress and defense (18.75%) followed by signaling (12.5%); and metabolism and structural compounds including secondary metabolite synthesis (12.5%). The categories of protein synthesis, folding and turnover; plant growth and development, and also undefined functions constituted 6.25% each.

A total of 51 DAPs (46.4%) were found to be common to all the three stages (*i.e.*, G0, G1, and G3). These belonged to functional categories like DAPs of unknown function (33.33%), signaling (15.68%); protein synthesis, folding and turnover (13.72%); stress and defense (11.76%), and membrane transport and trafficking (5.88%). The DAPs belonging to categories like photosynthesis, cell wall and cell structure; and also carbohydrate and energy constituted 3.92% whereas, those of plant growth and development, and detoxification constituted 1.96%.

When the DAPs of G0 and G1 were compared, six DAPs were common; and among these, four (66.7%) belonged to DAPs of unknown functions, while the remaining two (33%) belonged to membrane transport, trafficking, and energy. On the other hand, 13 DAPs were common to G0 and G3. In this case also, seven proteins of unknown functions were the most dominant category (53.85%) followed by two (15.38%) each of cell wall and cell structure; metabolism, structural compounds and secondary metabolite synthesis. Two DAPs belonging to each (7.69%) of signaling; and stress and defense were also recorded. A total of seven DAPs with unknown function; membrane transport, trafficking; and stress and defense categories were common to G1 and G3. These constituted 28.57%.

**Figure 4:**
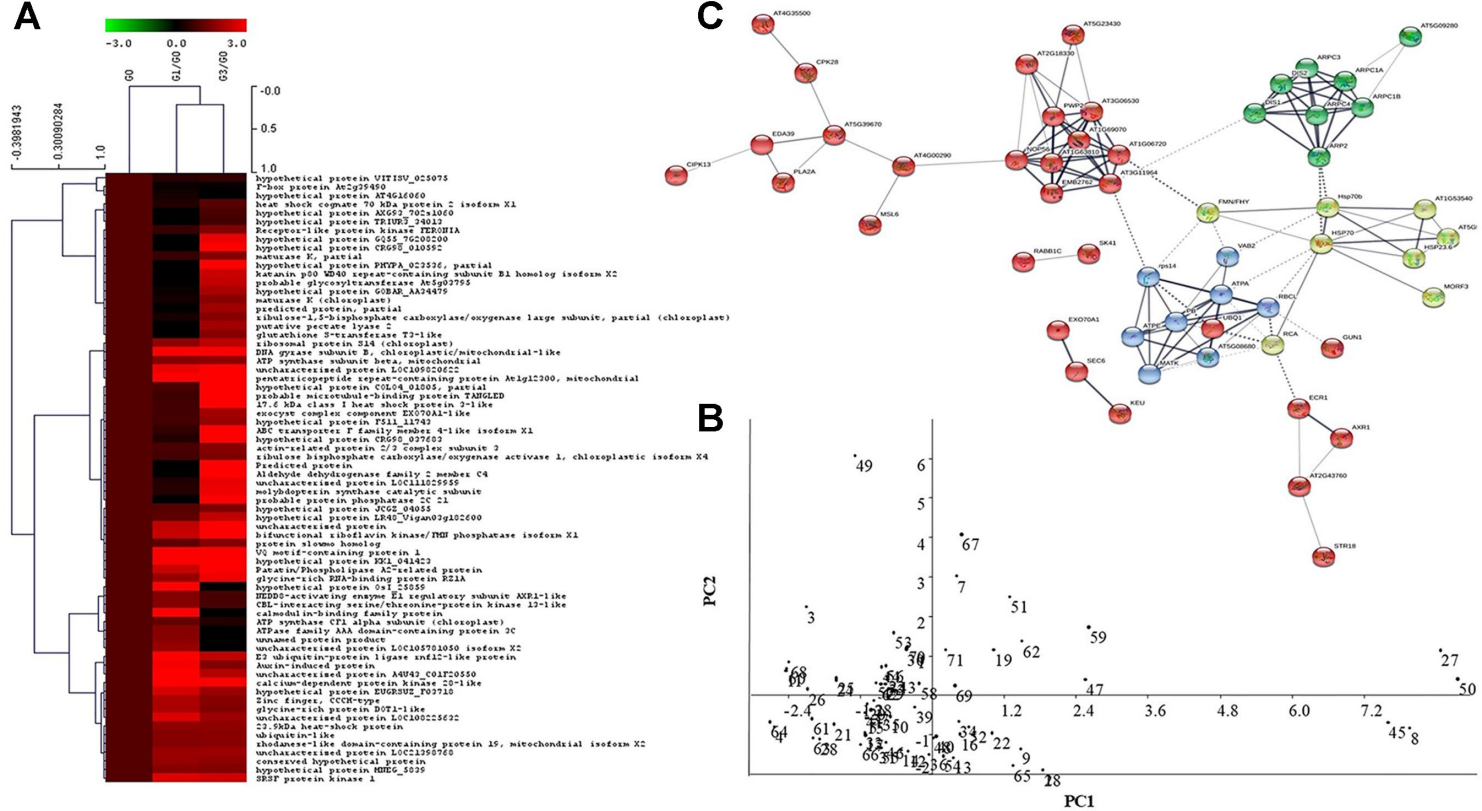
Analysis of proteomic data. **(A)** Heatmap showing different clusters of identified proteins and their relationships based on their functions and fold change. Heatmap representation and hierarchical clustering of protein dataset was performed by MeV software, wherein colour code represents the fold change of DAPs in G1 and G3 as compared to G0. **(B)** Principal component analysis showing the first two principal components of identified proteins (studied using Past software, version 3.18). Two PCs represent data reduction to whole dataset while allowing effective separation of samples. PC1 accounted for 73.75% and PC2 for 26.25% variance in score plot, respectively. **(C)** Protein-protein interaction showing a network of 53 unique protein homologs of *Arabidopsis* generated by string database. The network was analyzed and depicted in the form of nodes (proteins) and edges (association) in confidence view with thicker lines representing stronger association. Characterization of network proteins by Kmeans clustering revealed green, yellow, red, and blue coloured modules. The detailed information of the protein and their association can be found in **Supplementary Table S5**.

When 70 out of a total of 110 DAPs were subjected to hierarchical clustering and heatmap analysis (**Figure 4A**), five distinct cluster patterns were recorded. Of these, cluster one had three DAPs related to F-box and hypothetical proteins in both G1 and G3, whereas, cluster two comprised of 16 DAPs including those of protein synthesis and folding, defense related and heat shock proteins.These included the heat shock cognate 70 kDa protein 2 isoform X1; GLUTATHIONE S TRANSFERASE protein, cell wall synthesis, and structural proteins, photosynthetic protein (RUBISCO), signaling protein (FERONIA), structural compound related protein (PECTATE LYASE 2). Cluster three was comprised of 28 DAPs belonging to transport and trafficking, secondary metabolites and structural compounds, stress and defense related proteins such as 17.6 HEAT SHOCK PROTEIN and PATATIN, PENTATRICOPEPTIDE REPEAT, PHOSPHATASE 2 C PROTEIN of signaling and ACTIN RELATED PROTEIN 2/3 of cell wall synthesis and subunit of ATP SYNTHASE PROTEIN. Cluster four comprised of eight DAPs dominating in signaling and energy metabolism related proteins. Cluster five on the other hand, comprised of 15 DAPs, belonging to signaling, stress and defense, plant growth and development, detoxification, protein synthesis, folding and turnover in G1 and G3. In hierarchical clustering, clusters one and two separated out distinctly from clusters three, four, and five, thereby, revealing the relationships of the DAPs within clusters.

### Principal Component Analysis

The data in the present study were extracted in two principal components (**Figure 4B**). The first principal component, PC1 accounted for 73.75% and PC2 for a variance of 26.25% in score plot. Data reduction to whole dataset and their two dimensional representation allowed effective separation of samples. In PC1, two proteins namely, calcium dependent protein kinase 28 and hypothetical protein AXG93_702s1060 showed high positive correlation. In addition, a 17.6 kDa heat shock protein having potential adaptation to osmotic stress was extracted in PC1. In PC2, the VQ motif protein involved in signaling showed high positive correlation. Furthermore, the F-box protein, also known to play an important role in signaling, protein folding and turnover was extracted in PC2. The reduction data further revealed a distinct correlation of sucrose with signaling and also defense against stress. Several unannotated and hypothetical proteins were also extracted in PC1 which showed negative correlation with PC2 component.

### Protein Protein Interaction

When the 110 sucrose inducible DAPs obtained above were subjected to basic local alignment search tool (BLAST) against TAIR database, a total of 89 unique DAPs of *D. maximum* were found to be homologous to *Arabidopsis thaliana* (**Table S5**). In the PPI network and molecular interaction, 53 out of 89 homologs represented 110 DAPs based on neighbourhood, co-occurrence, co-expression, experimental evidences, text-mining, databases, and gene fusion. The illustrated PPI networks were visualized in confidence view in the form of nodes (proteins) and edges (association). Thicker lines symbolized strong association. Characterization of network proteins by Kmeans clustering revealed green, yellow, red, and blue colored modules (**Figure 4C**). Green colored module revealed a tight association between eight DAPs, and most of them were actin related proteins with probable involvement in the regulation of actin polymerization. These are known to play a critical role in the control of cell morphogenesis *via* the modulation of cell polarity. In the yellow colored module, two subunits of actin related protein interacted weakly with pectate lyase two protein on one side and ARP 2/3 complex protein and Hsp70 protein interaction on the other. In this module also, eight DAPs were tightly networked with each other and most of them were heat shock proteins. The HsP70 proteins interacted with actin related proteins (ARP2/3) on one side and bifunctional riboflavin kinase/FMN phosphatase protein, on the other. Furthermore, the HsP 70 of RNA polymerase II interacted with chloroplastic protein RIBULOSE BISPHOSPHATE CARBOXYLASE/OXYGENASE ACTIVASE 1, which in turn showed a weak interaction with blue colored module. The blue module had eight interacting DAPs comprised of five proteins of ATPase subunits and one each of 30s ribosomal protein, MATURASE K, and RUBISCO. The last two *i.e.*, RUBISCO and MATURASE K showed further interaction with RIBULOSE BISPHOSPHATE CARBOXYLASE/OXYGENASE ACTIVASE protein of yellow module. The red colored module four represented 29 DAPs from two different clusters. Cluster one of this module had 10 DAPs having strong association with each other. Most of these were ribonucleoproteins, proteins associated with synthesis and nucleotide processing, cell wall organization, uncharacterized proteins, and embryo defective protein. Ribonucleoprotein showed interaction with MECHANOSENSITIVE ION CHANNEL PROTEIN of Cluster two of the red module. This in turn interacted with calcium sensor protein and further with defense protein, PATATIN LIKE two on one side and signal transduction protein *i.e.*, calcium dependent protein kinase 28 on the other. This cluster also contained serine/threonine kinase and CBL INTERACTING signaling proteins. Interaction of one set of two proteins *i.e*. shaggy related protein and ras related proteins with each other on one side and interaction of a set of three transport and trafficking proteins on the other were observed. Interestingly, one ubiquitin protein of the red module interacted strongly with the blue color module.

### Real-Time-Polymerase Chain Reaction Validation of Some Key Differentially Abundant Proteins

**Figure 5:**
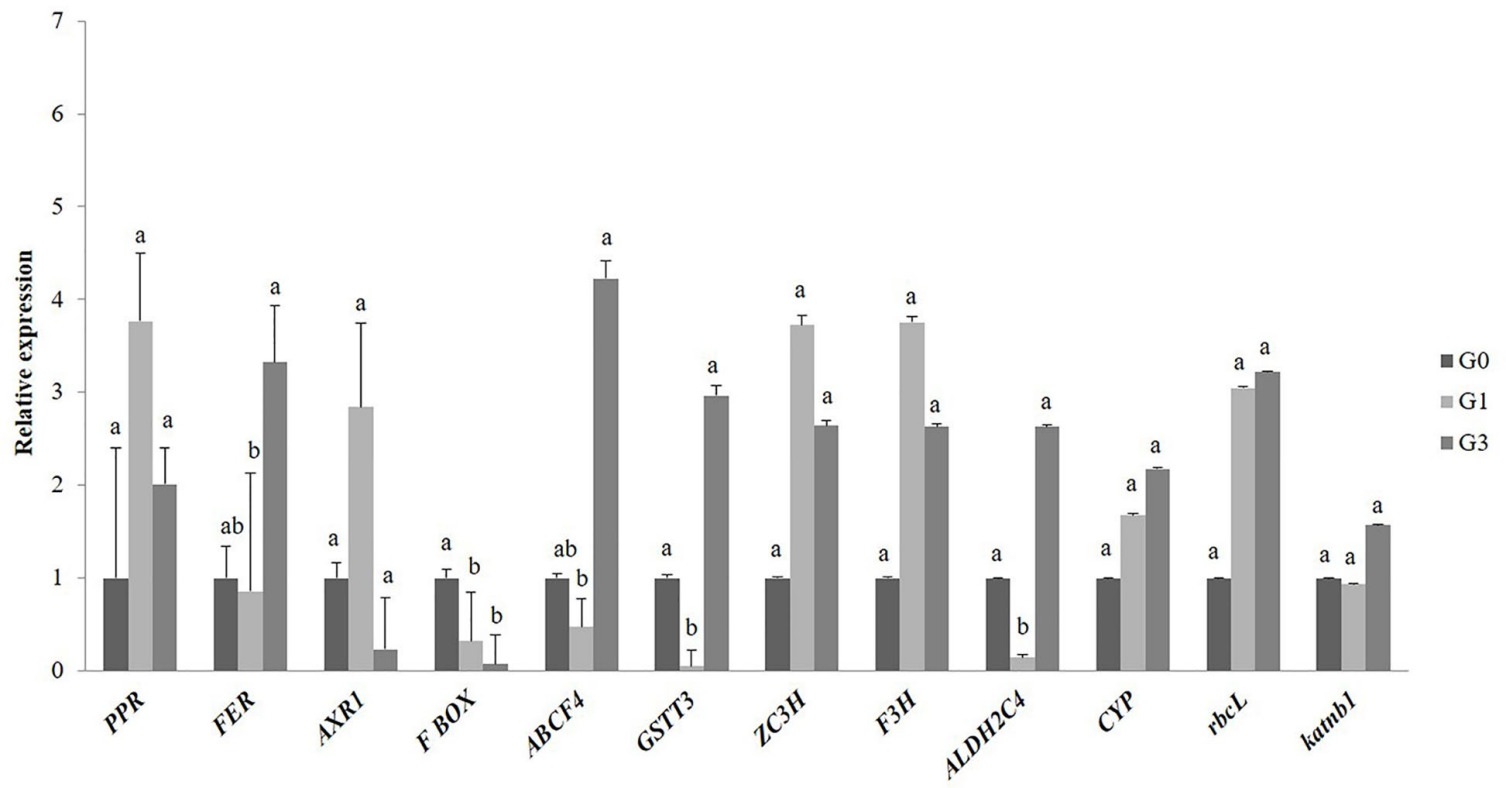
Quantitative real-time PCR validation of differentially abundant proteins of *Diplazium maximum* gametophytes. Transcript accumulation of genes encoding 12 proteins of G1 and G3 involved in signaling, stress, photosynthesis, transport, and cell wall synthesis is depicted. The expression levels of *F3H*, *ZC3H*, *rbcL*, *PPR*, *GSTT3*, *FER*, *CYP*, *ALDH2C4*, *katnb1*, *F-box*, *and AXR1* were normalized to the constitutive level of ELONGATION FACTOR 2 (*EF2*). The relative gene expression levels were finally calculated by the 2^−ΔΔt^ method. Error bars show the standard error (S.E.) for three biological replicates with each having three technical replicates. Different letters indicate the significant difference among the values at p ≤ 0.05.

The qRT-PCR expression profile of genes encoding some candidate DAPs of gametophytes validated our proteomics results. A total of 12 genes involved in signaling, cell wall synthesis, secondary metabolites synthesis, photosynthesis, stress and defense, transport and trafficking were studied. Out of these, four genes that appeared with similar patterns of up-regulation at 1 and 3% sucrose included *F3H* (secondary metabolite), *ZC3H* (stress responses), *rbcL* (photosynthesis), and *PPR* of pentatricopeptide repeat (signaling). Another four genes encoding transport protein (*ABCF4*), stress and defense proteins (*GSTT3*) and two genes encoding *FER* and *CYP* (signaling) were down-regulated at 1% but up-regulated at 3% sucrose. Gene homolog of aldehyde dehydrogenase protein, *ALDH2C4* and *katnb 1* of cell wall synthesis showed expression only at 3% sucrose. One gene encoding a protein of F-box family was down-regulated at 1 and 3% sucrose, whereas, *AXR 1* of signaling showed upregulation at 1% (**Figure 5**).

### Validation of Stress Response of Gametophytes to Sucrose Concentrations

#### Diameter, Moisture Content, Fresh and Dry Weights of Gametophytes

**Figure 6:**
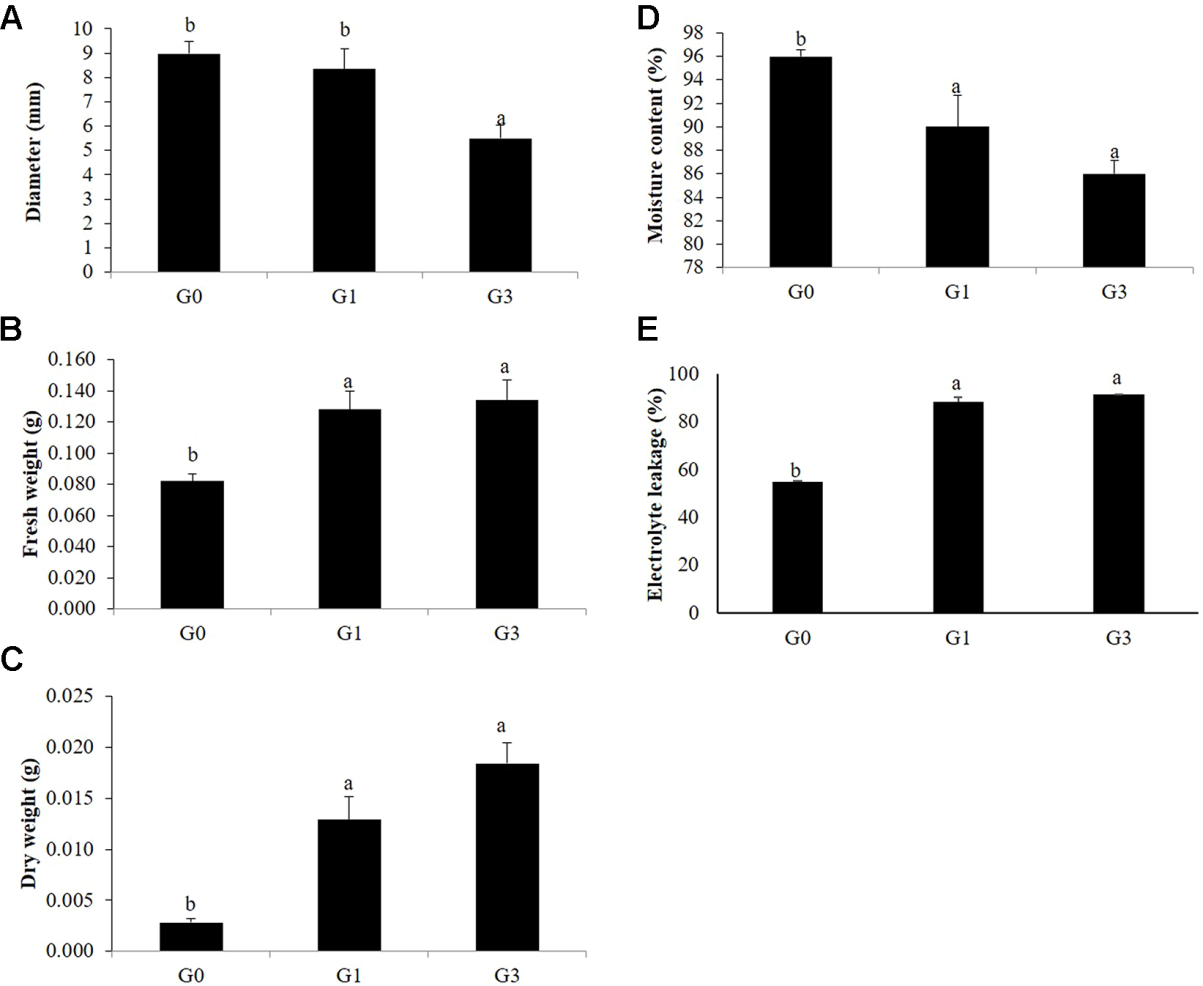
Morphological and physiological changes in gametophytes in response to sucrose concentrations. **(A)** Diameter. **(B)** Fresh weight. **(C)** Dry weight. **(D)** Moisture content. **(E)** Electrolyte leakage. Error bars show the standard error (S.E) for three biological replicates of each of G0, G1 and G3 with each having three technical replicates. Different letters indicate the significant differences among the values recorded for each parameter.

The diameter of the G0 gametophyte clumps was 8.96 mm after 30 days of incubation on sucrose free KM, the diameter reduced to 8.34 and 5.5 mm at 1 and 3% sucrose, respectively. The mean moisture content of G0 gametophytes was 97% but that of G1 and G3 were 90 and 86% at 1 and 3% sucrose, respectively. As compared to G0, significant increase in the fresh weights of G1 and G3 was recorded at 1 and 3% sucrose. However, the increase in G1 and G3 was at par. On the contrary, there was four and six folds increase in the dry weights of G1 and G3, respectively (**Figures 6A–D**).

#### Electrolyte Leakage

A significant difference was recorded between the EL of G0, G1, and G3. While the EL of G1 and G3 were 89.033 and 91.758%, respectively, that of G0 was only 55.434% (**Figure 6E**).

#### Detection of Reactive Oxygen Species

**Figure 7:**
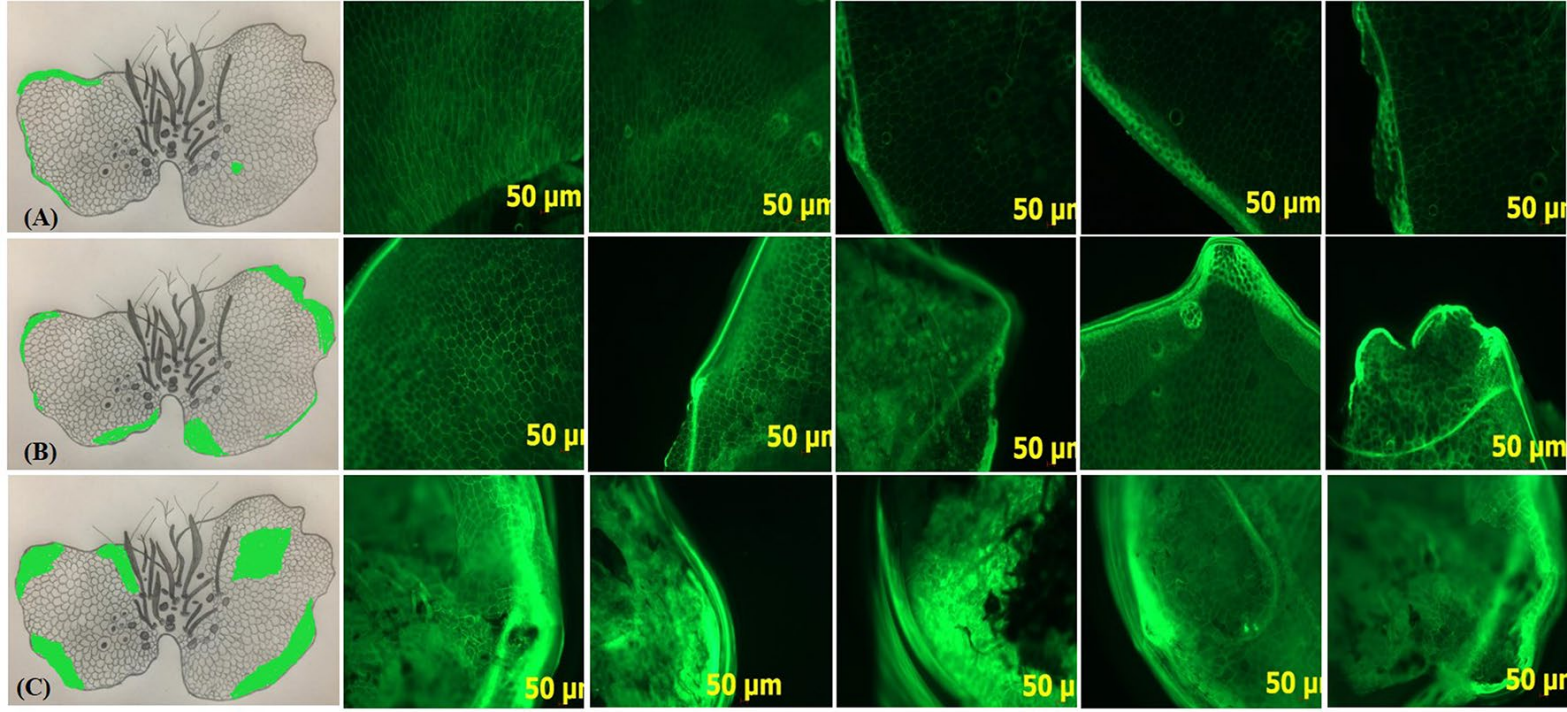
Accumulation of ROS in gametophytes in response to different sucrose concentrations. Fluorescence microscopy observations of DCFH-DA stained hand-cut transverse sections of G0, G1 and G3 at 5X magnification. Green fluorescent spots/layers in the sections **Ai-v, Bi-v,** and **Ci-v** show the localization of ROS at different regions of the prothallus. **A, B, C**, Pencil sketch diagrams of gametophytes depict the exact location of fluorescence on prothallial surface, where **(A)** G0 **(B)** G1 **(C)** G3.

Faint green fluorescent spots as well as a thin but bright green fluorescent layer were observed at the extreme margins of G0 prothallus. In comparison, there was a remarkable increase in the fluorescent intensity of the spots and the marginal layer in G1. In case of G3 however, the entire prothallus was intensely fluorescent (**Figure 7**).

#### Proline

**Figure 8:**
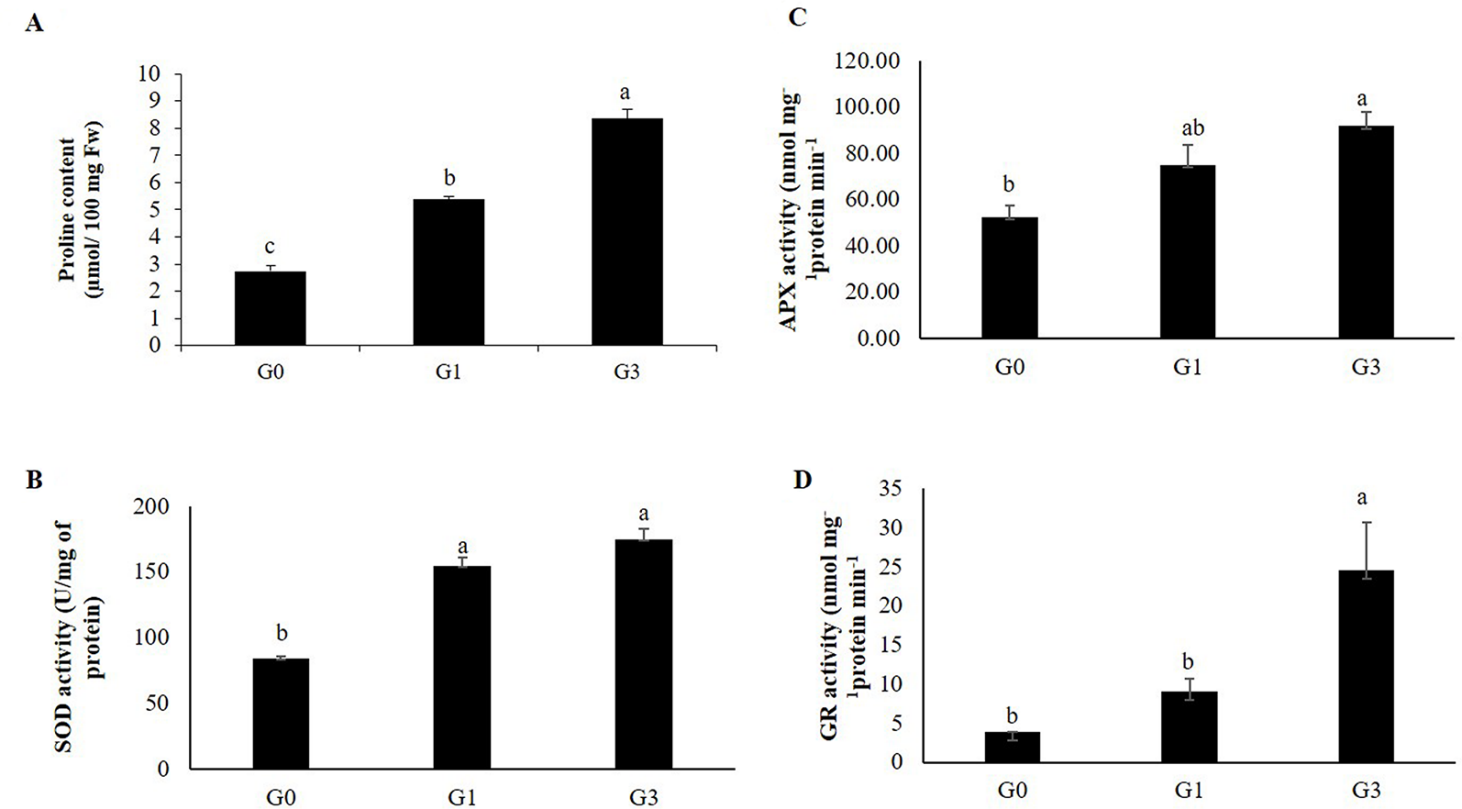
Changes in proline and ROS scavenging enzyme activity in gametophytes in response to sucrose concentration. **(A)** Proline content. **(B)** Superoxide dismutase (SOD). **(C)** Ascorbate peroxidase (APX). **(D)** Glutathione reductase (GR) activity. Error bars show the standard error (S.E.) for three biological replicates of each of G0, G1 and G3 with each having three technical replicates. Different letters indicate the significant differences among the values recorded for each parameter.

The proline content of G1 and G3 were 2.5 and 4.5 folds higher than that of G0 (**Figure 8A**).

#### Reactive Oxygen Species Scavenging Enzyme Activities

As compared to G0, there was significant increase in the activities of SOD, APX and GR in G1 and G3 (**Figures 8B–D**). The specific activity of SOD (U/mg protein) was 2.07 and 1.84 folds higher in G3 and G1, respectively. In case of APX, the specific activity was highest in G3 (91.79 nmol mg^−1^protein min^−1^) followed by G1 (75.0 nmol mg^−1^protein min^−1^) and G0 (52.5 nmol mg^−1^protein min^−1^). The GR activity showed a similar trend with highest activity in G3 (24.56 nmol mg^−1^protein min^−1^) followed by that in G1 (9.032 nmol mg^−1^protein min^−1^) and G0 (3.871 nmol mg^−1^protein min^−1^). Irrespective of the enzyme activity studied, highest activity was recorded in G3 and lowest in G0.

**Figure 9:**
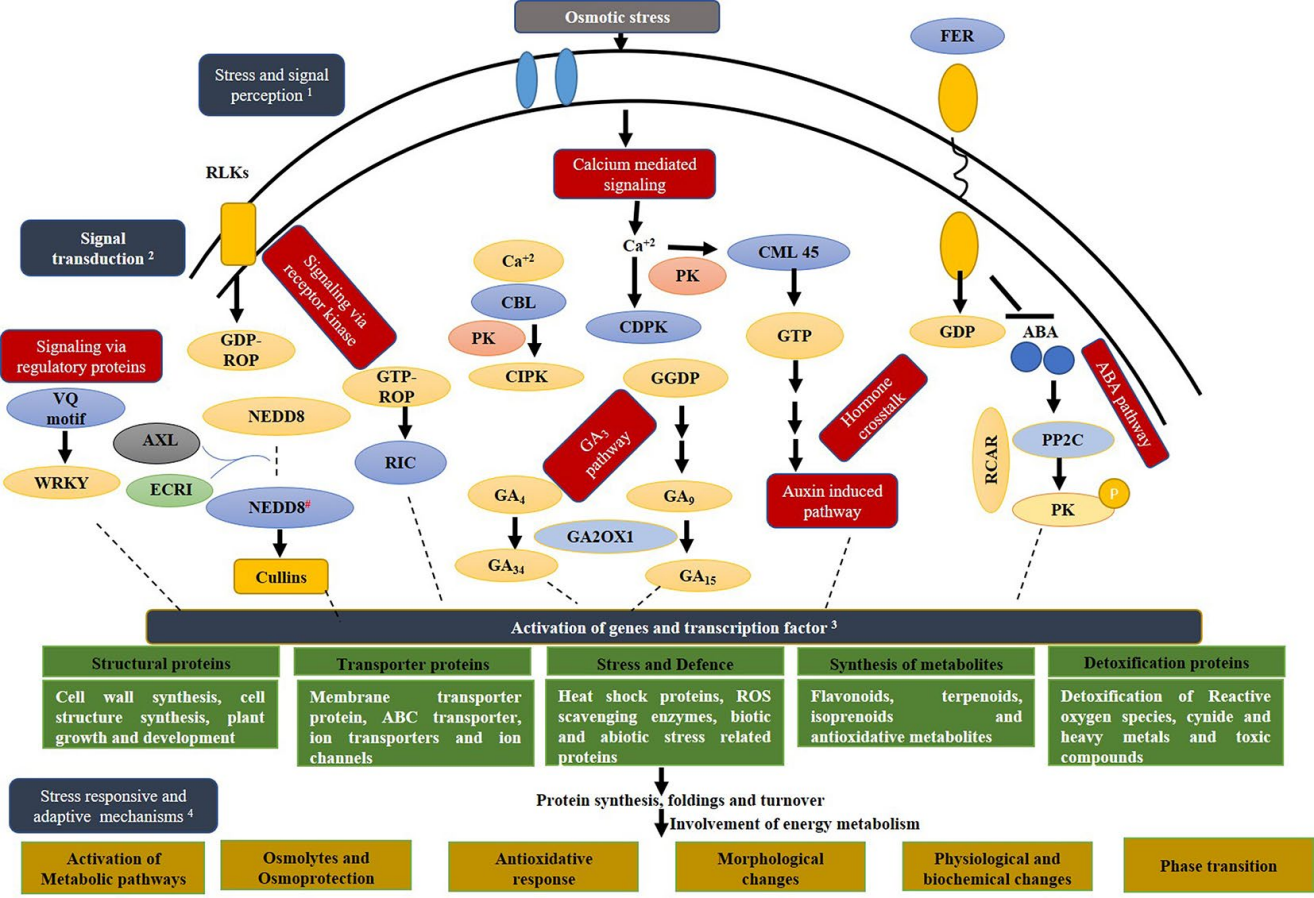
Hypothetical representation of the molecular basis of stress tolerance mechanisms operative in *Diplazium maximum* gametophytes in response to osmotic changes caused by sucrose concentrations. (1) Signal perception through receptors; (2) Transduction and signalling directly or through secondary messengers (red boxes), DAPs (blue ovals) and intermediates/products (yellow ovals); (3) Activation of genes, transcription factors and various categories of proteins (green boxes) and finally manifestation of (4) multiple mechanisms for stress tolerance (gold boxes). RLK: RECEPTOR LIKE KINASES; FER: RECEPTOR LIKE KINASE FERONIA; Ca ^+2^: Calcium ions; PK: protein kinase; CDPK, CALCIUM DEPENDENT PROTEIN KINASE; CBL, CALCINEURIN B LIKE PROTEIN; CIPK, CALCIUM INTERACTING SERINE/THREONINE PROTEIN KINASE; PP2C: PROBABLE PHOSPHATASE 2C; CML45: PROBABLE CALCIUM BINDING PROTEIN CML45; RIC11, CRIB DOMAIN CONTAINING PROTEIN RIC11 like; ABA, ABSCISIC ACID; GA2OX1, GIBBERELLIN 2 BETA-DIOXYGENASE 1-LIKE ISOFORM X1; VQ motif, VQ MOTIF-CONTAINING PROTEIN 1; NEDD8, NEDD8- ACTIVATING ENZYME E1 REGULATORY SUBUNIT AXR1-LIKE; NEDD*, activated NEDD8; AXL, AUXIN RESISTANT1-LIKE; ECR1, E1 C-TERMINAL RELATED.

All these observations led to a hypothetical mechanism underlying the response of a Polypodiales gametophyte to differential osmotic potentials created by 1 and 3% sucrose (**Figure 9**).

## Discussion

The gametophyte stage is a crucial phase in a fern’s life cycle (Banks, 1999; Quintanilla et al., 2007b; Pittermann et al., 2013). They have simple morphology that is devoid of vasculature and there is no risk of cavitation during drought stress. Thus, gametophytes are considered to be more tolerant to a stressful environment than the sporophytes (Sato and Sakai, 1979; Farrar, 1998; Watkins et al., 2007; Ebihara et al., 2013; Pittermann et al., 2013). Moreover, the mode of propagation (sexual or vegetative) in a gametophyte changes according to the availability of moisture and nutrients in their microenvironment with this facilitaties the successful establishment of the future sporophytic generation (Bell, 1992; Atallah and Banks, 2015). Obviously, these events entail sets of vital and novel genes that are differentially regulated under variable environmental conditions. However, nothing is known about the genes and proteins that are involved during the gametophytes response to micro-environmental changes.

In view of the above, the effect of 0, 1, 3, and 6% sucrose on *D. maximum* gametophytes was studied. Results revealed distinct changes in the morphology, growth, multiplication, and reproduction of the gametophytes (G1 and G3 as compared to G0) (**Figure 1**). Although the gametophytes multiplied into clumps at all concentrations of sucrose, the ones at 0 and 1% had loosely stacked prothalli, whereas, the ones at 3% were stacked closely into compact clumps. While there was no sporophyte development in G0 gametophyte, healthy sporophytes developed directly from the notch of individual G1 gametophytes or gametophytic clumps. The compact clumps of G3 supported only lanky and spindly sporophytes (**Figure 1I**).

**Figure 10:**
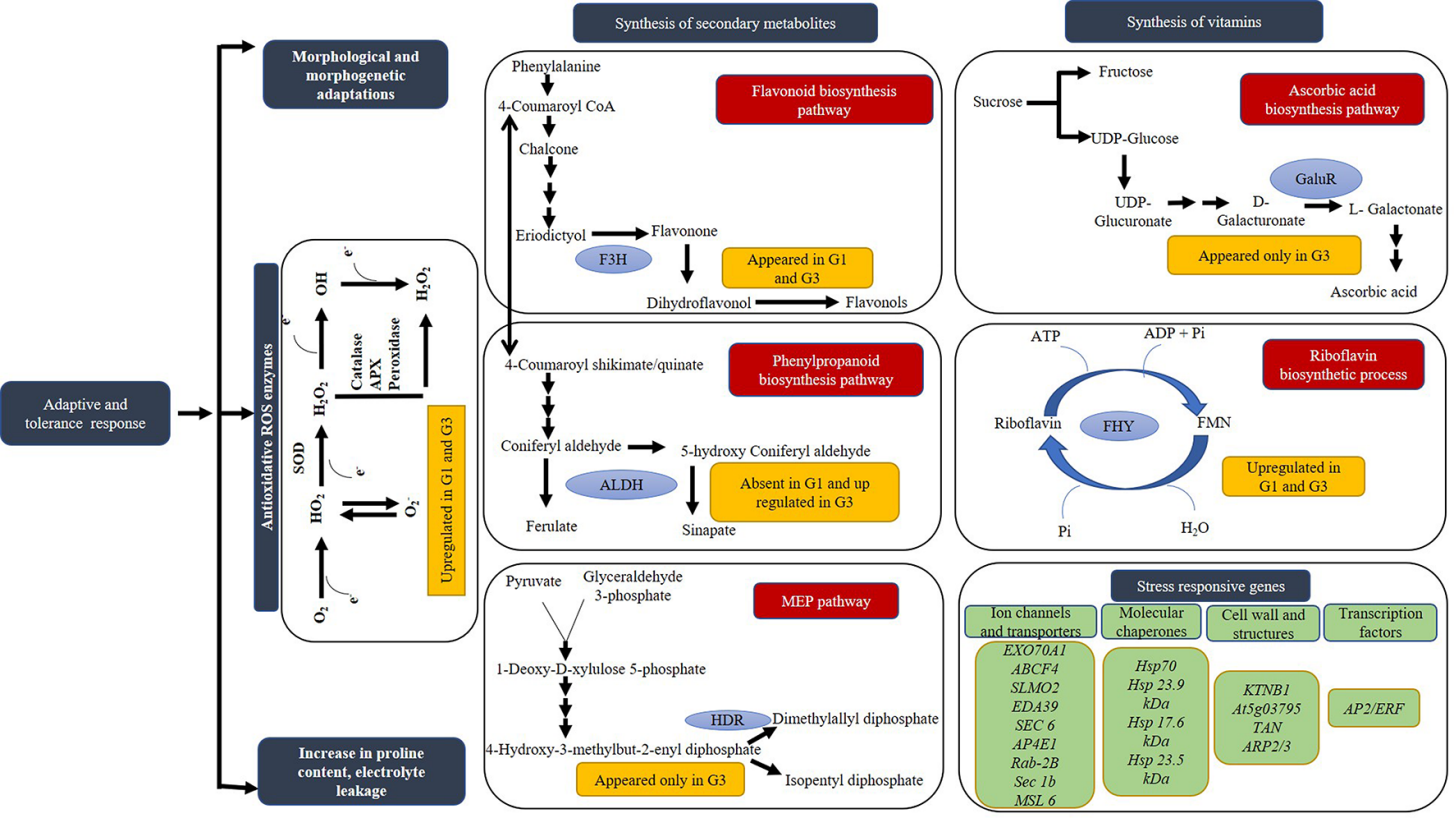
Schematic representation of the probable pathways and genes activated in response to sucrose inducible osmotic stress. The genes are depicted in green boxes, status of proteins in response to different concentrations of sucrose in gold boxes, mechanisms in blue boxes and pathways in red boxes. Based on proteins identified in *Diplazium maximum* gametophytes, biosynthesis of flavonoid, phenylpropanoid, ascorbic acid, riboflavin as well as MEP pathways are proposed to become operative in response to sucrose inducible osmotic stress. Blue ovals in different pathways depict the DAPs of metabolite synthesis such as F3H, FLAVANONE 3-HYDROXYLASE; ALDH, ALDEHYDE DEHYDROGENASE FAMILY 2 MEMBER C4; HDR, HYDROXY-3-METHYLBUT-2-ENYL DIPHOSPHATE REDUCTASE-LIKE ISOFORM X1; GaluR, D-GALACTURONATE REDUCTASE; FHY, BIFUNCTIONAL RIBOFLAVIN KINASE/FMN PHOSPHATASE.

Proteome analysis also revealed remarkable changes in the DAP profile of G0, G1 and G3. Various signaling pathways were found to be operative, wherein, 23.53 and 12.5% DAPs were recorded in G1 and G3 as compared to G0, respectively. The gametophytes appeared to perceive the signals caused due to changes in sucrose concentrations, and adjusted according to these changes in their micro-environment (**Figure 9**). Since a significant number of stress related DAPs were also recorded in G0, G1 and G3 (**Table 1**), it was assumed that changes created by sucrose were probably stressful for the *D. maximum* gametophytes (**Figure 10**). Reduction in diameter of gametophytes, decline in moisture content, and significant increases in EC and ROS at 3% as compared to 1% sucrose, but complete necrosis and death at 6% confirmed our assumption. Although 1 and 3% sucrose does not impose any stress on highly evolved tissues of higher plants, yet these concentrations of sucrose are surely stressful for the thin, delicate and simple gametophytes of ferns. Formation of ROS in a living cell is one of the first consequences of stress (Gill and Tuteja, 2010). Therefore, its higher accumulation in G1 and G3 at 1 and 3% sucrose was clearly indicative of stress in our study.

Apart from signaling and stress, DAPs involved in energy metabolism, protein synthesis modification and turnover, cell wall and cell structure synthesis, transport and trafficking, photosynthesis, plant growth and development, defense in response to stress and secondary metabolite synthesis were recorded in G1 and G3 as compared to G0 (**Table 1**). It was apparent from these DAPs that the gametophytes were making various adjustments for adaptation and defense against stressful changes in their microenvironment (**Figure 10**). Attempts were therefore, made to depict our understanding of these adaptations in a hypothetical mechanism. As per this hypothesis, five different signaling pathways were triggered after exposure of gametophytes to microenvironmental changes created by 1 and 3% sucrose. These were namely, (i) calcium dependent signaling pathway through the participation of CALCIUM DEPENDENT PROTEIN KINASE (CDPK28), CALMODULIN LIKE PROTEIN (CML45), CALCINEURIN B LIKE PROTEIN (CBL) and the CALCIUM INTERACTING PROTEIN KINASE (CIPK13), known for their involvement in plant development and stress responses; (ii) signaling *via* receptor like kinases (RLKs) through the participation of CRIB DOMAIN CONTAINING RIC11, SHAGGY RELATED PROTEIN KINASE KAPPA ISOFORM X3 and FERONIA, (iii) G protein signaling *via* RAS RELATED RAB1, (iv) hormonal signaling and cross-talk through PP2C21, AUXIN INDUCED PROTEIN and GA2OX1, and also (v) signaling through regulatory proteins such as VQ MOTIF and NEDD8-ACTIVATING ENZYME E1 REGULATORY SUBUNIT AXR1 LIKE (AXR1) (**Figure 9**).

In calcium signaling pathway, CDPK 28 was recorded in both G1 and G3. It is known for its role in stem elongation and vascular development (Schulz et al., 2013). While CBL and CIPK13 were abundant in G0 and G1, the CML45 of the CaM-sensor-protein-family appeared only in G3. CBL is known to respond to external stimuli and interact with the Ser/Thr kinases to form the CBL-CIPK interaction network for participation in the Ca^+2^ signaling pathway (Batistič and Kudla, 2009). On the other hand, the homologs of CMLs in *Arabidopsis* respond to biotic and abiotic stimuli and are involved in developmental processes (Magnan et al., 2008; Ranty et al., 2016).

The transmembrane proteins or the RLKs get phosphorylated in response to stress (Osakabe et al., 2013). Thus, there was appearance of RIC11 and SHAGGY related protein, uniquely in G1. The RLKs transmit signals through extracellular and intracellular domains (Shiu et al., 2004). The RIC11 protein transduces signals through GTP binding protein of Rho of Plants (ROP) family (Berken, 2006), whereas, the SHAGGY related protein is involved in kinase activity for determination of cell fate, patterning and cytokinesis (Dornelas et al., 1999). Homologs of SHAGGY related protein in *Arabidopsis* play an important role in growth and development of tissues and organs, particularly, gynoecium (Dornelas et al., 2000). FERONIA is another important receptor like protein kinase recorded in all the three stages G0, G1, and G3. FERONIA was validated through qRT-PCR analysis. The protein mediates mechanical/environmental stress induced signaling pathway and repairs injured cell walls. The protein is often required for cell elongation during vegetative growth (Shih et al., 2014). FERONIA also controls fertilization either directly or indirectly through hormonal cross-talks (Huck et al., 2003).

Member of the regulatory signaling *i.e.*, VQ MOTIF CONTAINING PROTEIN 1 in both G1 and G3, whereas, AXR1 in G1 were abundant. While the former modulates WRKY transcription factor (Lin and Jing, 2015), the latter is first activated by AXL-ECR1 under stress to initiate the signaling cascade. In addition, RAS RELATED RAB 1 appeared only in G1. It is a member of the G protein signaling pathway and plays vital role in transport, trafficking and stress inducible physiological and other processes of growth (Batoko et al., 2000).

Under hormonal signaling and crosstalk, the AUXIN INDUCED PROTEIN of auxin signaling and PP2C 21 were abundant in G1 and G3. The AUXIN INDUCED PROTEIN is an important regulator of gametophyte and sporophyte development in non-flowering plants (De Smet and Jurgens, 2007; Banks, 2009; Kato et al., 2015). Its abundance probably accounted for higher multiplication of G1 and G3 for closer proximity for sexual reproduction and sporophyte formation. The clade B protein, PP2C regulates the MAPK signaling pathway in fern allies like *Selaginella moellendorfii* (Fuchs et al., 2013).

The F box protein was recorded in both G1 and G3. Homologs of the protein are conserved in *Selaginella moellendorffii* and are known to participate in gibberellin signaling perception pathway. However, another protein, the GA2OX1 from gibberellin biosynthetic pathway not only determines sex in fern gametophytes through antheridiogen biosynthesis but also controls its mode of reproduction (Tanaka et al., 2014). Appearance of the protein only in G1 indicated a normal sexual mode of reproduction at 1% sucrose, while also accounting for the formation of healthy sporophytes. On the other hand, cytochrome P450 known to be responsible for apoximis in ferns (Grossmann et al., 2017) appeared only in G3 stage, wherein a large number of lanky sporophytes emerged from within the gametophyte clumps.

Activation of genes and transcription factors of signaling and transduction, transport and ion channels, cell wall and structural proteins, defense and chaperons coupled with energy metabolism, synthesis of metabolites, protein synthesis, and also modification and turnover must have led to cell wall alterations and physiological changes in G1 and G3 (**Figure 10**). These processes in turn appeared to be manifested as major changes in basic morphology and also increased biomass of G1 and G3 prothallus. Abundance of RIBULOSE BISPHOSPHATE CARBOXYLASE/OXYGENASE ACTIVASE 1 (RCA) and RIBULOSE-1,5-BISPHOSPHATE CARBOXYLASE/OXYGENASE LARGE SUBUNIT (RUBISCO) in G3 probably accounted for higher photosynthesis and increased biomass of photosynthetic prothalli at 3% sucrose. While RCA is involved in the activation of Rubisco *via* ATP dependent carboxylation, RUBISCO participates in carbon fixation (Salvucci and Ogren, 1996). The homolog of GLYCINE-RICH PROTEIN DOT1-like (DOT1) is known for its role in vascular tissue pattern formation and maintenance of cellular structure for adaptation under drought (Carmo et al., 2019). It was abundant in both G1 and G3 probably because sucrose at high concentrations is known to induce osmotic stress and drought like conditions. Even 1 and 3% sucrose was found to impose an adverse effect on *D. maximum* gametophytes in our study. Interestingly, the EPIDERMAL PATTERNING FACTOR-LIKE PROTEIN 2 (EPFL2) known for controlling stomatal patterning, growth, and pattern development in plants appeared only in G3 having tightly arranged clumps of prothalli (Caine et al., 2016).

A number of proteins known for their involvement in different stress responses and adaptation were differentially abundant in the *D. maximum* gametophytes. In addition to increased accumulation of proline, increased scavenging of ROS for enhanced tolerance to oxidative stress, DAPs of mostly, abiotic stress tolerance, secondary metabolite synthesis and detoxification were recorded at 1 and 3% sucrose in our study. Thus, the DAPs of 23.5 kDa heat shock protein (HSP), PHOSPHOPANTETHEINE ADENYLYLTRANSFERASE ISOFORM X1 and AP2/ERF domain-containing transcription factor appeared in G3 and proteins like ALDEHYDE DEHYDROGENASE 2 C4, GLUTATHIONE S TRANSFERASE T3 (GSTT3), 17.6 kDa class I HSP 3 and 23.9 kDa HSP were abundant in G3. Interestingly, RHODANESE LIKE DOMAIN CONTAINING PROTEIN 19, MITOCHONDRIAL ISOFORM X2 known for detoxification of ROS, heavy metals and cyanide, PATATIN/PHOSPHOLIPASE A2-RELATED PROTEIN, ZINC FINGER PROTEIN (ZC3H) and GLYCINE-RICH RNA-BINDING PROTEIN (RZ1A) known for their involvement in stress tolerance were abundant in both G1 and G3. However, PEROXIDASE 27 known for auxin catabolism, H_2_O_2_ scavenging and cell wall fortification and the RESTRICTED TEV MOVEMENT 2 protein, known for biotic stress tolerance appeared in G1 and G3. The qRT-PCR of *GSTT3* and *ZC3H* indicated an increase in stress response with increase in sucrose concentration in the culture medium.

It was evident from the appearance of DAPs of FLAVANONE 3-HYDROXYLASE (F3H) of flavonoid biosynthetic pathway in both G1 and G3, that specific secondary metabolite pathways became operative in *D. maximum* gametophytes under stress. Even the qRT-PCR of *F3H* in G1 and G3 supported our assumption. The appearance of 4-HYDROXY-3-METHYLBUT-2-ENYL DIPHOSPHATE REDUCTASE-LIKE ISOFORM X1 (HDR) of MEP pathway in G3 only, indicated the requirement of terpenoids biosynthesis under high stress condition.

In addition to above, DAPs of vitamins, hormones, and cofactor biosynthetic pathways were recorded (**Table 1**). BIFUNCTIONAL RIBOFLAVIN KINASE/FMN PHOSPHATASE known to drive the hydrolysis of FMN to riboflavin and also phosphorylation of riboflavin to FMN was abundant in G1 and G3. Moreover, D-GALACTURONATE REDUCTASE (D-GaluR), an intermediate of ascorbic acid biosynthetic pathway appeared only in G3. Similarly, MOLYBDOPTERIN SYNTHASE CATALYTIC SUBUNIT of cofactor biosynthetic pathway was abundant in G3.

In heatmap analysis, there was abundance of the heat shock cognate 70 kDa in cluster 2 and the 17.6 kDa class I heat shock protein 3 in cluster 3 (**Figure 4A**). These indicated an important role of heat shock proteins in the adaptation of *D. maximum* gametophytes to micro-environmental changes. While heat shock cognate 70 kDa is known to facilitate the adaptation and survival of eukaryotic organisms under various environments (McCallister et al., 2015), the 17.6 kDa class I heat shock protein 3 imparts osmo-tolerance to sudden changes in a eukaryotic organism’s environment (Sun et al., 2001). The PPI network revealed the proteins involved in signaling, structural alterations, stress tolerance, and photosynthesis in gametophytes subjected to changes created by 1 and 3% sucrose in the culture medium (**Figure 4C**). Again, in PCA I and II components, proteins of signaling and heat shock were enriched, further indicating that the gametophytes responded to 1 and 3% sucrose in their culture medium by triggering multiple mechanisms of adaptation (**Figure 4B**).

## Conclusion

Our study revealed that the gametophytes of *D. maximum* are highly sensitive to stress. Yet the gametophytes are equipped with a battery of defense mechanisms that help them adapt to any change in their environment. Multiple mechanisms were found to become operative immediately after perception of signals of environmental changes (stress due to sucrose concentration in this case) by the gametophytes. This then led to differential abundance of various proteins involved in morphogenesis and adaptation. Five different signal transduction pathways were triggered. This was followed by increased scavenging of ROS, increased accumulation of proline, synthesis of secondary metabolites (as evident from aldehyde dehydrogenase family 2 member C4, Flavanone 3-hydroxylase, and 4-hydroxy-3-methylbut-2-enyl diphosphate reductase-like isoform X1) higher rate of vegetative growth resulting in significant increase in prothallus biomass and finally changes in basic morphology from thin and delicate prothalli to gametophyte clumps of higher biomass. These changes ensured higher surface area for photosynthesis, closer proximity for sexual reproduction and finally, transition towards sporophyte. This was further supported by the appearance of proteins involved in transport and trafficking in both G1 and G3 but those of plant growth and development in G3 only (**Supplementary Table S4**). Besides providing insights into the molecular basis of adaptive changes in *D. maximum* gametophytes, several useful proteins (HEAT SHOCK PROTEINS, PATATIN/PHOSPHOLIPASE A2-RELATED PROTEIN, RESTRICTED TEV MOVEMENT 2), phytoremediation and environment protection (RHODANESE LIKE DOMAIN-CONTAINING PROTEIN 19) were identified in the study.

## Data Availability Statement

Mass spectrometric output files, MASCOT search results and Peak list corresponding to proteomic analysis were deposited in Proteome Xchange Consortium *via* the PRIDE partner repository with the data set identifier ‘PXD014763’.

## Ethics Statement

Human Subject Research: No human studies are presented in this manuscript. Animal Subjects: No animal studies are presented in this manuscript. Human Images: No potentially identifiable human images or data is presented in this study.

## Author Contributions

BS, PT executed the experimental work and analyzed the results. BS contributed in experimental design and writing of manuscript. RJ performed MALDI-TOF and PDQuest analysis. AB supervised the work, conceptualized and wrote the manuscript. All authors read and approved the manuscript.

## Conflict of Interest

The authors declare that the research was conducted in the absence of any commercial or financial relationships that could be construed as a potential conflict of interest.

## Supplementary Material

Table S1 List of contaminant peaks.

Table S2 Primer sequences for qRT PCR analysis.

Table S3 Peptide Sequences and MS spectra.

Table S4 List of differentially abundant proteins (DAPs) of *D. maximum* gametophytes that appeared or disappeared in response to differential osmotic potential created by 1 and 3% sucrose (G1 and G3) as compared to no sucrose (G0).

Table S5 Tair annotation of identified proteins and their description for protein protein interaction network

Figure S1 Three biological replicates of 2-DE gels from the gametophytes of *D. maximum*. The gels were stained with Coomassie Brilliant Blue G-250. Molecular weight (MM) in kDa and pI of proteins are indicated on the left and top of the gels, respectively. (A1, B1, C1) G0 (A2, B2, C2) G1 (A3, B3, C3) G3.

## References

[B1] Alongi D. A. Hill J. P. Germino M. J. (2009). Opportunistic heterotrophy in gametophytes of the homosporous fern *Ceratopteris richardii* . Botany 87, 799–806. 10.1139/B09-039

[B2] Atallah N. M. Banks J. A. (2015). Reproduction and the pheromonal regulation of sex type in fern gametophytes. Front. Plant Sci. 6, 100. 10.3389/fpls.2015.00100 25798139 PMC4351586

[B3] Banks J. A. (1999). Gametophyte development in ferns. Annu. Rev. Plant Physiol. Plant Mol. Biol. 50, 163–186. 10.1146/annurev.arplant.50.1.163 15012207

[B4] Banks J. A. (2009). Selaginella and 400 million years of separation. Annu. Rev. Plant Biol. 60, 223–238. 10.1146/annurev.arplant.59.032607.092851 19575581

[B5] Bates L. S. Waldren R. P. Teare I. K. (1973). Rapid determination of free proline for water stress studies. Plant Soil 39, 205–208. 10.1007/BF00018060

[B6] Batistič O. Kudla J. (2009). Plant calcineurin B–like proteins and their interacting protein kinases. Biochim. Biophys. Acta 1793, 985–992. 10.1016/j.bbamcr.2008.10.006 19022300

[B7] Batoko H. Zheng H. Q. Hawes C. Moore I. (2000). A rab1 GTPase is required for transport between the endoplasmic reticulum and Golgi apparatus and for normal Golgi movement in plants. Plant Cell 12, 2201–2218. 10.1105/tpc.12.11.2201 11090219 PMC150168

[B8] Beauchamp C. O. Fridovich I. (1971). Superoxide dismutase: improved assays and an assay applicable to acrylamide gels. Anal. Biochem. 44, 276–287. 10.1016/0003-2697(71)90370-8 4943714

[B9] Bell P. R. (1992). Apospory and apogamy: implications for understanding the plant life cycle. Int. J. Plant Sci. 153, S123–S136. 10.1086/297070

[B10] Berken A. (2006). ROPs in the spotlight of plant signal transduction. Cell Mol. Life Sci. 63, 2446–2459. 10.1007/s00018-006-6197-1 16932855 PMC11136380

[B11] Bradford M. M. (1976). A rapid and sensitive method for the quantitation of microgram quantities of protein utilizing the principle of protein-dye binding. Anal. Biochem. 72, 248–254. 10.1016/0003-2697(76)90527-3 942051

[B12] Caine R. S. Chater C. C. Kamisugi Y. Cuming A. C. Beerling D. J. Gray J. E. (2016). An ancestral stomatal patterning module revealed in the non-vascular land plant *Physcomitrella patens* . Development 143, 3306–3314. 10.1242/dev.135038 27407102 PMC5047656

[B13] Carlberg I. Mannervik B. (1985). Glutathione reductase assay. Methods Enzymol. 113, 484–495. (Orlando FL Academic. 10.1016/S0076-6879(85)13062-4 3003504

[B14] Carmo L. S. Martins A. C. Martins C. C. Passos M. A. Silva L. P. Araujo A. C. (2019). Comparative proteomics and gene expression analysis in *Arachis duranensis* reveal stress response proteins associated to drought tolerance. J. Proteomics 192, 299–310. 10.1016/j.jprot.2018.09.011 30267876

[B15] Cooke T. J. Racusen R. H. (1988). The growth forms of developing fern gametophytes: a theoretical consideration of form and function relationships. Ann. Bot. 62, 633–641. 10.1093/oxfordjournals.aob.a087703

[B16] De Smet I. Jurgens G. (2007). Patterning the axis in plants–auxin in control. Curr. Opin. Genet. Dev. 17, 337–343. 10.1016/j.gde.2007.04.012 17627808

[B17] DeSoto L. Quintanilla L. G. Méndez M. (2008). Environmental sex determination in ferns: effects of nutrient availability and individual density in *Woodwardia radicans* . J. Ecol. 96, 1319–1327. 10.1111/j.1365-2745.2008.01425.x

[B18] Dornelas M. C. Wittich P. Recklinghausen I. Lammeren A. Kreis M. (1999). Characterization of three novel members of the *Arabidopsis* SHAGGY-related protein kinase (ASK) multigene family. Plant Mol. Biol. 39, 137–147. 10.1023/A:1006102812280 10080716

[B19] Dornelas M. Lammeren A. Kreis M. (2000). *Arabidopsis thaliana* SHAGGY-related protein kinases (AtSK11 and 12) function in perianth and gynoecium development. Plant J. 21, 419–429. 10.1046/j.1365-313x.2000.00691.x 10758494

[B20] Ebihara A. Yamaoka A. Mizukami N. Sakoda A. Nitta J. H. Imaichi R. (2013). A survey of the fern gametophyte flora of Japan: frequent independent occurrences of non-cordiform gametophytes. Am. J. Bot. 100, 735–743. 10.3732/ajb.1200555 23510760

[B21] Farrar D. R. (1998). The tropical flora of rockhouse cliff formations in the eastern United States. J. Torrey Bot. Soc 125, 91–108. 10.2307/2997297

[B22] Fuchs S. Grill E. Meskiene I. Schweighofer A. (2013). Type 2C protein phosphatases in plants. FEBS J. 280, 681–693. 10.1111/j.1742-4658.2012.08670.x 22726910

[B23] Ghawana S. Paul A. Kumar H. Kumar A. Singh H. Bhardwaj P. K. (2011). An RNA isolation system for plant tissues rich in secondary metabolites. BMC Res. Notes 4, 85. 10.1186/1756-0500-4-85 21443767 PMC3079660

[B24] Gill S. S. Tuteja N. (2010). Reactive oxygen species and antioxidant machinery in abiotic stress tolerance in crop plants. Plant Physiol. Biochem. 48, 909–930. 10.1016/j.plaphy.2010.08.016 20870416

[B25] Greer G. McCarthy B. (1999). Gametophytic plasticity among four species of ferns with contrasting ecological distributions. Int. J. Plant Sci. 160, 879–886. 10.1086/314188 10506469

[B26] Grossmann J. Fernández H. Chaubey P. M. Valdés A. E. Gagliardini V. Cañal M. J. (2017). Proteogenomic analysis greatly expands the identification of proteins related to reproduction in the apogamous fern *Dryopteris affinis* Ssp. Affinis. Front. Plant Sci. 8, 336. 10.3389/fpls.2017.00336 28382042 PMC5360702

[B27] Huck N. Moore J. M. Federer M. Grossniklaus U. (2003). The Arabidopsis mutant feronia disrupts the female gametophytic control of pollen tube reception. Development 130, 2149–2159. 10.1242/dev.00458 12668629

[B28] Kato H. Ishizaki K. Kouno M. Shirakawa M. Bowman J. L. Nishihama R. (2015). Auxin-mediated transcriptional system with a minimal set of components is critical for morphogenesis through the life cycle in *Marchantia polymorpha* . PloS Genet. 11, e1005084. 10.1371/journal.pgen.1005084 26020919 PMC4447296

[B29] Kaur D. Dogra V. Thapa P. Bhattacharya A. Sood A. (2015). *In vitro* flowering associated protein changes in *Dendrocalamus hamiltonii* . Proteomics 15, 1291–1306. 10.1002/pmic.201400049 25475561

[B30] Knop W. (1865). Quantitative Untersuchungüber die Ernährungsprozesse der Pflanzen. LandwirtschVersStn. 30, 292–294.

[B31] Laemmli U. K. (1970). Cleavage of structural proteins during the assembly of the head of bacteriophage T4. Nature 227, 680–684. 10.1038/227680a0 5432063

[B32] Lin R. Jing Y. (2015). VQ-motif-containing protein family of plant specific transcriptional regulators. Plant Physiol. 169, 371–378. 10.1104/pp.15.00788 26220951 PMC4577417

[B33] Lutts S. Kinet J. M. Bouharmont J. (1996). Effects of salt stress on growth, mineral nutrition and proline accumulation in relation to osmotic adjustment in rice (*Oryza sativa* L.) cultivars differing in salinity resistance. Plant Growth Reg. 19, 207–218. 10.1007/BF00037793

[B34] Magnan F. Ranty B. Charpenteau M. Sotta B. Galaud J. P. Aldon D. (2008). Mutations in AtCML9, a calmodulin-like protein from *Arabidopsis thaliana* alter plant responses to abiotic stress and abscisic acid. Plant J. 56, 575–589. 10.1111/j.1365-313X.2008.03622.x 18643966

[B35] McCallister C. Siracusa M. C. Shirazi F. Chalkia D. Nikolaidis N. (2015). Functional diversification and specialization of cytosolic 70-kDa heat shock proteins. Sci. Rep. 5, 9363. 10.1038/srep09363 25791537 PMC4366816

[B36] Mehra P. N. (1972). Some aspects of differentiation in cryptogams. Res. Bull. Panjab Univ. 23, 221–242.

[B37] Nakano Y. Asada K. (1981). Hydrogen peroxide is scavenged by ascorbate specific peroxidase in spinach chloroplasts. Plant Cell Physiol. 22, 867–888. 10.1093/oxfordjournals.pcp.a076232

[B38] Osakabe Y. Yamaguchi-Shinozaki K. Shinozaki K. PhanTran L. S. (2013). Sensing the environment: key roles of membrane-localized kinases in plant perception and response to abiotic stress. J. Exp. Bot. 64, 445–458. 10.1093/jxb/ers354 23307915

[B39] Osundeko O. Davies H. Pittman J. K. (2013). Oxidative stress tolerant microalgae strains are highly efficient for biofuel feedstock production on wastewater. Biomass Bioenerg. 56, 284–294. 10.1016/j.biombioe.2013.05.027

[B40] Page C. N. (2002). Ecological strategies in fern evolution: a neopteridological overview. Rev. Palaeobot. Palynol. 119, 1–33. 10.1016/S0034-6667(01)00127-0

[B41] Pajaron S. Pangua E. Quintanilla L. G. Jimenez A. (2015). Influence of water availability on gender determination of gametophytes in a diploid–polyploid complex of a xerophytic fern genus. AoB Plants 7, plv047. 10.1093/aobpla/plv047 25940203 PMC4480211

[B42] Pittermann J. Brodersen C. Watkins J. E. (2013). The physiological resilience of fern sporophytes and gametophytes: advances in water relations offer new insights into an old lineage. Front. Plant Sci. 4, 285. 10.3389/fpls.2013.00285 23935601 PMC3733004

[B43] Qui Y. L. Taylor A. B. McManus H. A. (2012). Evolution of the life cycle in land plants. J. Syst. Evol. 50, 171–194. 10.1111/j.1759-6831.2012.00188.x

[B44] Quintanilla L. G. DeSoto L. Jiménez A. Méndez M. (2007b). Do antheridiogens act *via* gametophyte size? A study in *Woodwardia radicans* (Blechnaceae). Am. J. Bot. 94, 986–990. 10.3732/ajb.94.6.986 21636467

[B45] Racusen R. H. (2002). Early development in fern gametophytes: interpreting the transition to prothallial architecture in terms of coordinated photosynthetic production and osmotic ion uptake. Ann. Bot. 89, 227–240. 10.1093/aob/mcf032 12099354 PMC4233796

[B46] Ranty B. Aldon D. Cotelle V. Galaud J. P. Thuleau P. Mazars C. (2016). Calcium Sensors as Key Hubs in Plant Responses to Biotic and Abiotic Stresses. Front. Plant Sci. 7, 327. 10.3389/fpls.2016.00327 27014336 PMC4792864

[B47] Salvucci M. E. Ogren W. L. (1996). The mechanism of Rubisco activase: insights from studies of the properties and structure of the enzyme. Photosyn. Res. 47, 1–11. 10.1007/BF00017748 24301702

[B48] Sato T. Sakai A. (1979). Freezing resistance of gametophytes of the temperate fern *Polystichum retroso-paleaceum* . Can. J. Bot. 58, 1144–1148. 10.1139/b80-141

[B49] Sato T. Sakai A. (1980). Cold tolerance of gametophytes and sporophytes of some cool temperate ferns native to Hokkaido. Can. J. Bot. 59, 604–608. 10.1139/b81-085

[B50] Sato T. (1982). Phenology and wintering capacity of sporophytes and gametophytes of ferns native to northern Japan. Oecologia 55, 53–61. 10.1007/BF00386718 28309902

[B51] Schuettpelz E. Pryer K. M. (2009). Evidence for a Cenozoic radiation of ferns in an angiosperm-dominated canopy. Proc. Natl. Acad. Sci. U. S. A. 106, 11200–11205. 10.1073/pnas.0811136106 19567832 PMC2708725

[B52] Schulz P. Herde M. Romeis T. (2013). Calcium-dependent protein kinases: hubs in plant stress signaling and development. Plant Physiol. 163, 523–530. 10.1104/pp.113.222539 24014579 PMC3793034

[B53] Shih H. W. Miller N. D. Dai C. Spalding E. P. Monshausen G. B. (2014). The receptor-like kinase FERONIA is required for mechanical signal transduction in Arabidopsis seedlings. Curr. Biol. 24, 1887–1892. 10.1016/j.cub.2014.06.064 25127214

[B54] Shiu S. H. Karlowski W. M. Pan R. Tzeng Y. H. Mayer K. F. Li W. H. (2004). Comparative analysis of the receptor-like kinase family in Arabidopsis and rice. Plant Cell 16, 1220–1234. 10.1105/tpc.020834 15105442 PMC423211

[B55] Sigel E. M. Schuettpelz E. Pryer K. M. Der J. P. (2018). Overlapping patterns of gene expression between gametophyte and sporophyte phases in the fern *Polypodium amorphum* (Polypodiales). Front. Plant Sci. 9, 1450. 10.3389/fpls.2018.01450 30356815 PMC6190754

[B56] Suetsugu N. Mittmann F. Wagner G. Hughes J. Wada M. (2005). A chimeric photoreceptor gene, NEOCHROME, has arisen twice during plant evolution. Proc. Natl. Acad. Sci. U. S. A. 102, 13705–13709. 10.1073/pnas.0504734102 16174755 PMC1224637

[B57] Sun W. Bernard C. Van De Cotte B. Van Montagu M. Verbruggen N. (2001). At-HSP 17.6A, encoding a small heat-shock protein in Arabidopsis, can enhance osmotolerance upon overexpression. Plant J. 27 (5), 407–415. 10.1046/j.1365-313X.2001.01107.x 11576425

[B58] Suo J. Zhao Q. Zhang Z. Chen S. Cao J. G. Liu G. (2015). Cytological and proteomic analyses of *Osmunda cinnamomea* germinating spores reveal characteristics of fern spore germination and rhizoid tip growth. Mol. Cell. Proteomics 14, 2510–2534. 10.1074/mcp.M114.047225 26091698 PMC4563732

[B59] Tanaka J. Yano K. Aya K. Hirano K. Takehara S. Koketsu E. (2014). Antheridiogen determines sex in ferns *via a* spatiotemporally split gibberellin synthesis pathway. Science 343, 463–473. 10.1126/science.1259923 25342803

[B60] Testo W. Sundue M. (2016). A 4000-species dataset provides new insight into the evolution of ferns. Mol. Phylogenet. Evol. 105, 200–211. 10.1016/j.ympev.2016.09.003 27621129

[B61] Valledor L. Menéndez V. Cañal M. Revilla M. Fernández H. (2014). Proteomic approaches to sexual development mediated by antheridiogen in the fern *Blechnum spicant* L. Proteomics 14, 2061–2071. 10.1002/pmic.201300166 25044718

[B62] Watkins J. E. Cardelús C. L. (2012). Ferns in an Angiosperm World: cretaceous radiation into the epiphytic niche and diversification on the forest floor. Int. J. Plant Sci. 173, 695–710. 10.1086/665974

[B63] Watkins J. E. Jr. Mack M. C. Sinclair T. R. Mulkey S. S. (2007). Ecological and evolutionary consequences of desiccation tolerance in tropical fern gametophytes. New Phytol. 176, 708–717. 10.1111/j.1469-8137.2007.02194.x 17822408

