# Supplementary material for: Proteome Analysis of the Gametophytes of a Western Himalayan Fern Diplazium maximum Reveals Their Adaptive Responses to Changes in Their Micro-Environment
Source: Front Plant Sci. 2019 Dec 17;10:1623. doi: 10.3389/fpls.2019.01623 (PMC6928197; doi:10.3389/fpls.2019.01623)
Supplement: Table S1 — List of contaminant peaks. [file Table_1.docx]

**Supplementary Table**

**Table S1 List of contaminant peaks**

| **Trypsin Peaks** |
| --- |
| 842.5099  1045.5642  2239.1359 |
| **CHCA matrix cluster signals** |
| 833.09  855.07  871.04  883.06  893.03  899.03  946.95  1044.11  1082.07  1271.09  1287.08 |
