# Supplementary material for: Proteome Analysis of the Gametophytes of a Western Himalayan Fern Diplazium maximum Reveals Their Adaptive Responses to Changes in Their Micro-Environment
Source: Front Plant Sci. 2019 Dec 17;10:1623. doi: 10.3389/fpls.2019.01623 (PMC6928197; doi:10.3389/fpls.2019.01623)
Supplement: Table S2 — Primer sequences for qRT PCR analysis. [file Table_2.docx]

**Table S2 Primer sequences for qRT PCR analysis**

| **Sr. no** | **Gene** | **Primer sequence** | **Primer length** |
| --- | --- | --- | --- |
| 1 | *PPR F* | AACAGCCCCTACCTTGATACCTT | 24 |
| 2 | *PPR R* | GTGACCTATGTGTGCATCCTGAA | 24 |
| 3 | *FER F* | TGCCACCCTTCTTGGATCTT | 20 |
| 4 | *FER R* | TCACTGCTTTGGCCGTCATA | 21 |
| 5 | *AXR1 F* | GGCCATGTCCTCAGCATATAAAGT | 25 |
| 6 | *AXR 1 R* | GCTGCACTATCATTTAGAACTCTTTGAA | 28 |
| 7 | *F box F* | CAGGTCGCCCTCACTTATGC | 21 |
| 8 | *F box R* | CTACTTTCTTGGTCGCCTTGAGA | 23 |
| 9 | *ABCF4 F* | GAGGGATTACAGTGTGGAGTTTCATT | 26 |
| 10 | *ABCF4 R* | TCATCTCGACCGGGATAGCT | 20 |
| 11 | *GSTT3 F* | AGGGCTACCCACGGATCAC | 19 |
| 12 | *GSTT3 R* | TTGCCCAATTTGCTCCTCAT | 20 |
| 13 | *ZC3H F* | CGATGTTTCAGTACGATTCTTCCA | 24 |
| 14 | *ZC3H R* | CGCTTCAGAACTCTTTCACGAA | 22 |
| 15 | *F3H F* | TCTTCTCACCTCTAACCCTCCAA | 23 |
| 16 | *F3H R* | CCACTCCCAAGCTGGCTTT | 19 |
| 17 | *ALDH2C4 F* | GGCAGCCTCCATCACTATTCTC | 22 |
| 18 | *ALDH2C4 R* | CGCTAGCCACATGGACATTG | 21 |
| 19 | *CYP F* | GGCGAACAGCATCCTCTTTG | 21 |
| 20 | *CYP R* | TTTCGGAAGTTGCTGGAGAAG | 22 |
| 21 | *rbcL F* | CAGCGAAAGCGAGTCTGAATAG | 23 |
| 22 | *rbcL R* | GGGTGTTACCCCACCTTCATC | 22 |
| 23 | *Katnb 1 F* | GAGTTTTGTTTGCTCTCCTCTTGAA | 26 |
| 24 | *Katnb 1 R* | AGGACCCGTCTGGCAAGTC | 20 |
