# Supplementary material for: Proteome Analysis of the Gametophytes of a Western Himalayan Fern Diplazium maximum Reveals Their Adaptive Responses to Changes in Their Micro-Environment
Source: Front Plant Sci. 2019 Dec 17;10:1623. doi: 10.3389/fpls.2019.01623 (PMC6928197; doi:10.3389/fpls.2019.01623)
Supplement: Table S3 — Peptide Sequences and MS spectra. [file Table_3.docx]

**Table S3 Peptide Sequences and MS spectra**

| **Protein name** | **Number of peptides** | **Peptide sequences** | **MS spectra** |
| --- | --- | --- | --- |
| **Signaling** | | | |
| Receptor-like protein kinase FERONIA | 10 | TLGTDK  DFAVALLHHSQK  DFAVALLHHSQKK  LNQGSQQGLHEFR  HTIIHRDVK  TTNILLNEK  VGPTSESK  THVSTAVK  SGWGAAMSTRR  SNDVCDESTTSYDSK |  |
| NEDD8-activating enzyme E1 regulatory subunit AXR1-like | 11 | MATESK  MATESK  YDRQLR  CVCAFLQELNDAVK  LDKICR  EFKDLIK  SEADCLAMELRVR  NFCK  NFCK  NFCKNAR  FTANYNR |  |
| CBL-interacting serine/threonine-protein kinase 13-like | 15 | FMEGLLGRIITK  EITPESPR  VIDKEMIVK  HPYTVHLLEVMATK  HPYTVHLLEVMATKTK  IYIVMEYVR  LREGTAR  GLFRCPK  WFSPELKELMNR  FYIENDKLCR  DWSVRLEGCR  LEGCR  GPLTIKVEIFELATSLVVVEVK  GGFIEEYEEFCNK  LMHYQADEVEEAMC |  |
| VQ motif-containing protein 1 | 6 | SSGK  DVVQK  LTGKDSR  VAYEMQEPNNIVAK  NISFK  LFREMPPVEDFWID |  |
| Calcium-dependent protein kinase 28-like | 10 | VAVKK  YSEK  VAAECHLHGLVHRDMKPENFLFK  AVDFGLSDFIKPGKK  RPFWDK  RPFWDKTENGIFK  QFALR  ALASTLDEEELSDLK  VHTGLK  TASMINPA |  |
| Auxin-induced protein | 7 | FALYYK  YIGVSETSPEDIRR  AHAVHPLSAVQLEWSLWTR  ELGIGIVAYSPLGR  KSFP  YNEAAMK  VSVQR |  |
| Pentatricopeptide repeat-containing protein At1g12300, mitochondrial | 14 | MMLR  MLRTQR  NLSYKEK  QMELNGIAHNIYTLNIMINCFCRR  LGFAFSAMGKIFK  MMDK  GCPADQFTYGPILNRMCK  MEHR  DMIR  LDEANQMLDLMVSK  LDEANQMLDLMVSK  LNVAK  LLLRK  AHLRDGDVSISVELIEEMK |  |
| Probable protein phosphatase 2C 21 | 6 | VAMYCAK  SVCSR  CVLSR  QKLR  LRSGR  CLPPR |  |
| SRSF protein kinase 1 | 7 | GGYHAVRVGDSFSGGR  VGDSFSGGR  YIAQR  VREICK  RAAAR  ISGRR  GGEGFSEDEDHLAMK |  |
| Gibberellin 2-beta-dioxygenase 1-like isoform X1 | 9 | MVVLSKQTTEQYAYIR  HCNKTSAFSSTGIIPVVDLSKPEAK  EKAGPPNPFGYGNK  FRNVLNDYICAVR  KMGCEILELMAEGLNIEEK  ENEESLYK  EFTWFEYKK  LADNR  LGHFEIFSAS |  |
| Shaggy-related protein kinase kappa isoform X3 | 14 | MASSGLGHGGAGSSR  MASSGLGHGGAGSSR  SSNGFKGSSSSVDWLGK  GSSSSVDWLGK  CQETGEIVAIKK  IARQYSR  MNQR  MNQRMPLIYVK  MPLIYVK  ALAYIHNYIGICHR  GEPNVSYICSR  CMNPNYTEFK  FFQYSPNLR  SQGIPRDIVHR |  |
| CRIB domain-containing protein RIC11-like | 5 | AMKMK  GIYKSFK  SLLTTSSSTDFDQRSSQPVISVRPR  YSSSTSSPNPSYHLQNLQDQRVSK  YSFK |  |
| Ras-related protein RABB1c-like | 10 | SYAYLFKYIVIGDTGVGK  YIVIGDTGVGK  SITRSYYR  SYYR  GAAGALLVYDITRR  ETFNHLVSWLEDAR  QHANANMTIMLIGNKSDLAHR  EHGLVFMEASAK  TAQNVEEAFVR  TAATIFKK |  |
| Probable calcium-binding protein CML45 | 7 | SSSPIKK  FDSEDLSGLFGEDEEVVAMDDEVK  ELQRVLCLLGMK  VLCLLGMK  EGSEVQR  DMIR |  |
| **Stress and Defence** | | | |
| Heat shock cognate 70 kDa protein 2 isoform X1 | 26 | TTPSYVAFTDTER  TTPSYVAFTDTERLIGDAAK  NQVAMNPINTVFDAKR  LIGRR  NAVVTVPAYFNDSQR  DAGVIAGLNVMR  IINEPTAAAIAYGLDK  IINEPTAAAIAYGLDKK  ATAGDTHLGGEDFDNR  LRTACER  TLSSTAQTTIEIDSLYEGIDFYSTITR  ARFEELNMDLFR  CMEPVEKCLR  CLRDAK  MDKSTVHDVVLVGGSTR  STVHDVVLVGGSTR  NTTIPTKK  EQVFSTYSDNQPGVLIQVYEGER  EQVFSTYSDNQPGVLIQVYEGERTR  TRTR  TRDNNLLGK  TTGQKNK  ITITNDK  ITITNDKGR  NALENYSYNMR  DEKISSK |  |
| 23.9kDa heat-shock protein | 6 | SGAPVAFR  YDDDDDDDDYSGR  LLSLMEDVASQTGGLSSTAGAGASRLGR  VPMPGLTK  VPMPGLTKEHVEVR  MDKIK |  |
| Patatin/Phospholipase A2-related protein | 7 | SMMGPK  EKLGETK  IFCSYEVK  SDPCK  EILR  ENLENLVK  DMRSPSR |  |
| 17.6 kDa class I heat shock protein 3-like | 6 | EEKNEK  VERGK  GKFTR  LPQNAK  AAMENGVLTVTIPKVPEK  KPATK |  |
| Zinc finger, CCCH-type | 7 | KCTR  CPRR  GSCSR  TEACK  FSDR  FSDRLAR  TESCGMTQLGNGSSSYK |  |
| Glutathione S-transferase T3-like | 10 | MATSISVAR  ATSISVAR  LSRSAALMEEESLALLK  DCIVGNK  QRYR  GHGKK  QSRMTFSEFQSMWSLK  MTFSEFQSMWSLK  MTFSEFQSMWSLK  LSKIK |  |
| Glycine-rich RNA-binding protein RZ1A | 6 | LQFRCR  LSSSR  TTKEGLR  NAFEK  AIEGMHGK  FLDGR |  |
| Protein RESTRICTED TEV MOVEMENT 2 | 8 | YGKLK  FENGR  KQEDEK  QEDEKR  VAGAVK  HEEGEKGDAVPDVQK  GDAVPDVQK  KEAVLEPAAAGNGR |  |
| Peroxidase 27-like | 9 | FVLKFFLPISIMLLLSGSADAQGLK  GYGVIDTIKAK  AKLEK  GPSWEVPTGR  ASLIAETRQLPAPIFTFQQLK  LYNFTGK  ASNPR  DFGVSMVNMGNTGVLIGK  KNCAVVN |  |
| Phosphopantetheineadenylyltransferase isoform X1 | 7 | AENK  AENKQSPVNEMEK  AENKQSPVNEMEK  QSPVNEMEKGPHSYGAVVLGGTFDR  RLLK  IVVGICTGPMLVK  IEAKK |  |
| 23.5 kDa heat-shock protein | 7 | MASAVDCK  SGAPVAFR  LLSLMEDVASQTGGLSSTAGAGASR  VPMPGLTK  VPMPGLTK  YNRR  MDKIK |  |
| AP2/ERF domain-contasining transcription factor, partial | 7 | IICHDPDATDSSSDEEEEQIDKSK  TNTNTNTK  LGDNKR  DPFKK  MIAAEK  SSVEKVR  ECNVGK |  |
| **Transport and Trafficking** | | | |
| Exocyst complex component EXO70A1-like | 6 | MMYYR  YYRR  DEGASR  ESRFK  FNMQFDELHQRQSQWTVPDTELR  SFLKR |  |
| ABC transporter F family member 4-like isoform X1 | 12 | SEASVEKK  QESDK  VDADKMDVDEGQVK  GDEEGK  LDEDEDEDEGKVEK  EKFDK  LMEFCDILDIPVAK  LMEFLEAPHATTSELLAEKEQSSK  TESASK  ESDDESEEEEKPKK  TEAAKEK  KPASK |  |
| Protein slowmo homolog | 11 | VRGYK  VTAATWR  VDGATGVLESTR  VDGATGVLESTRLVSVNAPCPWWLQR  SSVDAAAR  SSVDAAARTMQIVTR  SSVDAAARTMQIVTR  TMQIVTR  NVTLKDFVEVEEK  QEMNIR  FQQNSAKGR |  |
| Calmodulin-binding family protein | 11 | HELFGLR  SMRLK  TNMER  IREAR  LLVPEKLNNLVIEK  SYRTR  ALDFAALK  WARAR  SELQHQCIRYLGPK  YSIDSGESSDGASFK  WTSGVGPRIGCVR |  |
| AP-4 complex subunit epsilon | 16 | SDNYLIVCAALNAVCR  AIMALHR  SPSSVSHLFSNFR  DLVVSFVSILK  GYDYHQMPAPFIQIR  LASEKMYTVVGDIFK  YMGIDALGRLIK  MTKSSNVEVIVDR  MIDYMISINDNHYK  MIDYMISINDNHYKTYIASR  LIAEGFGEDDDAADSQLRSSAVESYLR  YSASYITGK  IYAFEIASGR  IYAFEIASGRK  VDMLPECQSLVEELSASHSTDLQQR  VDMLPECQSLVEELSASHSTDLQQR |  |
| Exocyst complex component SEC6 isoform X8 | 11 | KSTTAMASSK  DKCYEK  CYEK  CYEKIR  VQPPK  VQPPKR  QRLAEPASEIGLEPLCAIINNNLR  EYVSVNK  HPATIQPEVVER  EAIPR  EAIPRK |  |
| Ras-related protein Rab-2-B | 6 | MITIDNKPIK  SYYR  GAAGALLVYDITRR  ETFNHLASWLEDARQHANANMTVMLIGNK  AVSYEEGEQFAK  ENGLVFMEASAKTAQNVEEFIK |  |
| Putative protein transport Sec1b | 9 | LGCGGEMSMSGADFNGVGEDPRIFR  MAEITDAGVSLVEDLFK  MAEITDAGVSLVEDLFKR  IGALR  MNTFVSK  LPSDDMKAINSLR  FDAQK  NSDDSQSSDSSVLRHASGDFK  IFVFIVGGATR |  |
| Mechanosensitive ion channel protein 6-like | 24 | ISAGGGGTEISHKELPILLDHEPAAVHR  GYGVEVEDPPMK  QKLR  ELQDEYKGQQSPWQQSHHER  HGSPTISGVQNDCAAEAAR  CTSNLSFQR  SGPLR  SGPLRSGFLGK  SGFLGK  NFLLR  RVLVSLLVSTLIWLVK  KNEEAEER  NEEAEER  SSHGKSSHGK  EIRSER  FMREDEVLR  NWVVNAFRER  TIFEAIIFLFVMHPFDVGDR  QRIISYIEGNK  IISYIEGNK  AHWSPSPMFVFKDVEELNR  WARR  VCQELDIQYR  LLPIDINVHSLPSSAPSMGFTST |  |
| **Protein synthesis, folding and turnover** | | | |
| F-box protein At2g39490 | 12 | EKENTSK  EIIGRIVSFLPNESSMETILISTR  LLLDFSPLK  LNNQQK  LNNQQKISYK  ISYKSFTCNIFSLK  DEYCMDALFSFLK  SYSTGR  SYSTGRSVSWFMQATK  LEHVNLIKFMGFTSR  FMGFTSR  FMGFTSRK |  |
| Maturase K, partial | 9 | LYPLLFK  KSSSLIVK  LVPSLEGK  TISVFSKK  TYFYRK  GTPLLLNK  KSLYR  LSCAR  SFLKR |  |
| Ubiquitin-like | 4 | MQIFVKTLIGK  QIFVK  ESTIHLVLSLCGGYREDEVYPIIIR  EDEVYPIIIRTYK |  |
| E3 ubiquitin-protein ligase rnf12-like protein | 6 | MMNQVVR  NQVVR  NQVVRGLNTR  GSTMMQR  SSTRR  STCPLCR |  |
| Maturase K (chloroplast) | 8 | EEFQR  LILLEK  NSISIFSKR  SILSSK  KSLYR  YVLR  LSCAR  SFLKR |  |
| Ribosomal protein S14 (chloroplast) | 6 | TLVKK  RQLK  DSGLSR  HVLR  HVLREMAHTCLLPGSTK  EMAHTCLLPGSTKSSW |  |
| DNA gyrase subunit B, chloroplastic/mitochondrial-like | 8 | GLEPVR  WLNTDK  MIKDK  AALAAK  TSSLPGKLADCSSTNPEESEIFIVEGDSAGGSAK  IIILTDADVDGAHIR  VERGK  QLVVEDAAEANVVFSSLMGRVDVR |  |
| Protein RMD5 homolog A | 11 | MELDSLR  VIEKR  ASSSAK  ELNVALSK  GSRDEALK  AEIQK  LTTVMAAKK  QSIMK  QSIMK  QSIMKLSK  QLHF |  |
| Protein RNA-directed DNA methylation 3-like | 19 | SASKTPTIWK  LISSSELEEIRPLIKNR  EWLSELFGEQK  EWLSELFGEQKK  HIYR  GGELLSSGANDVSSSSKSYLLPAK  SYLLPAKSTK  ISGVPGKGK  YNLGGKDVIFTVGQSLK  DVIFTVGQSLKIR  IQRK  VDGQFKMGR  MGRGDGQFNMGK  GGNMGR  GSNNQFDMGR  EHFVMGR  GGKEQFNMGR  TGTGGSRGGR  GQSSGWNGQDNK |  |
| E3 ubiquitin protein ligase DRIP2 | 9 | MMLGQVVKVQK  KCIYMK  CIYMK  VIGAESNSTSSPSGSVKPRR  KYLVQK  YLVQK  IQTTVGSSAK  KVSLQK  VSLQKHLATAAEFHLLQQ |  |
| **Metabolism and synthesis of metabolites** | | | |
| Aldehyde dehydrogenase family 2 member C4 | 18 | MTLEVKFTK  AFDHGK  GRIMMK  IMMKYADLIDQHK  IMMKYADLIDQHK  IMMKYADLIDQHK  VVDIEGSADMLRYYAGAADK  MAGNLHGYTLR  KIMEAAAK  KIMEAAAK  IMEAAAK  FVTKVTESMK  SWVVGDPFDPHVHQGPQVDK  AQFEK  AQFEKVLSYIEHGK  VLSYIEHGK  REGATLVTGGKPCGEK  YLQVK |  |
| Molybdopterin synthase catalytic subunit | 9 | DTFDGK  STWMLHKIAVAHR  IAVAHR  ADALEACK  ENTEFLDR  ENTEFLDRR  VEDEELK  ATAEDK  ATAEDK |  |
| Bifunctional riboflavin kinase/FMN phosphatase isoform X1 | 9 | SCFCK  EREENK  LGLTQK  DYQLPLTPDQFIKEINPLYIER  FGKPAPDLFEETAK  KIDVWLIDANTDLTSEQK  MQICLVGYIRSWDNK  LHEQK  LGSMEMDK |  |
| Putative pectate lyase 2 | 8 | NVIDK  DIEKYK  YKVTDLFDDPLSPK  GKVWITFK  AQPPSIVMGPNAK  MDGDAIR  MDGDAIRLVTAR  VMLLGHDDGHLRDK |  |
| Flavanone 3-hydroxylase | 11 | EFFALPPEEKLR  FDMSGGKK  EIVTYFSYPIR  LLEVLSEAMGLEK  ACVDMDQK  HTDPGTITLLLQDQVGGLQATRDGGK  IREGEKPILEEPITFADMYK  IREGEKPILEEPITFADMYK  EGEKPILEEPITFADMYKR  LAKEK  KLLQDQQDIEK |  |
| Galacturonate, partial | 10 | PVIGMGTSSYPR  AAILEAIR  HFDTAFAYGSEQDLGEAIAEALRLGLINSR  DELFITTKLWASSAEK  LWASSAEKDLVVPSIK  NLQLEYIDMYIIHWPFK  LGLAR  DFCKAK  VLGSDVIEEIALAR  WVYEQGVSIV |  |
| 4-hydroxy-3-methylbut-2-enyl diphosphate reductase-like isoform X1 | 10 | NTSLQR  NMGSEFSCMIK  IVYR  KNLDSEETHWCVIFELVCR  TTVEGYVKNVTPK  GCFIMLSR  GCFIMLSRK  TSGSGR  VHECVKAK  IDEERR |  |
| **Energy metabolism** | | | |
| ATPase family AAA domain-containing protein 3C | 13 | LCSAAAIAAAFTSMSMSQNR  AQMLR  IATEQQIQAQQR  LTEEQNRR  TLLTDR  ESSMGR  ESSMGR  SHKAPFR  IHEIFDWAK  LLKLYLNK  LYLNK  YLMGEDK  KTEGFSGR |  |
| ATP synthase CF1 alpha subunit (chloroplast) | 20 | ATGKIAQIPVSDSFLGR  IAQIPVSDSFLGR  VVNALAQPIDGKGQIPASEFR  LIESPAPGIISR  LIESPAPGIISRR  SVYEPLQTGLIAIDSMIPIGR  ELIIGDR  ELIIGDRQTGK  ASSVAQVVDTFQDR  QAQAYR  QMSLLLR  RPPGR  EAYPGDVFYLHSR  EAYPGDVFYLHSRLLER  LLER  AMKQVAGK  QVAGKLK  TTQNQLAR  LRELLK  EYVITNKPQFGEITR |  |
| ATP synthase subunit beta, mitochondrial | 13 | ISPSTTSR  VAEYATSAAATAPPSPPPPAK  LVLEVAQHLGENMVR  TIAMDGTEGLVRGR  VVDLLAPYQR  AHGGFSVFAGVGER  EGNDLYREMIESGVIK  CALVYGQMNEPPGAR  VGLTGLTVAEHFR  FTQANSEVSALLGR  IPSAVGYQPTLATDLGGLQER  QISELGIYPAVDPLDSTSR  MTVARAR |  |
| ATP synthase beta subunit, partial (chloroplast) | 14 | ISPSTTSR  VAEYATSAAATAPPSPPPPAK  LVLEVAQHLGENMVR  TIAMDGTEGLVRGR  VVDLLAPYQR  AHGGFSVFAGVGER  EGNDLYREMIESGVIK  CALVYGQMNEPPGAR  VGLTGLTVAEHFR  FTQANSEVSALLGR  IPSAVGYQPTLATDLGGLQER  QISELGIYPAVDPLDSTSR  MTVARAR |  |
| Cytochrome P450 | 8 | MGLNVGAWILLFLSSLALLSLIK  FLHK  FIHGNTKEIAAMK  EIAAMK  GIEDLIMETIK  GKIMAVDPDTADFLGQLVK  LGNLVIPANVILHVPVLALHHDR  NCVGSNFATNTAKITLAMILQR |  |
| **Cell wall and cell structure** | | | |
| Katanin p80 WD40 repeat-containing subunit B1 homolog isoform X2 | 19 | LWDLEEAK  TLTGHR  GCIHTYKGHTR  GVNAIRFTPDGR  VFSWEPIR  ETKTLGR  ESKVLSSAGSVPGTPQR  ATEVSCVADR  ATEVSCVADRNTFAAVK  EDIFTVSGK  EDIFTVSGKSGTMSMSESPASYEDER  DGYAMESQKR  LQAVYRYWER  YWERNDVK  IERCNR  CFIELEKVK  VKCCLPTLMR  CCLPTLMR  SAQELNLALQDV |  |
| Probable glycosyltransferase At5g03795 | 11 | MDQTR  MDQTR  DQTRVSR  MKDMLQK  DMLQK  LMENSKR  LMENSKR  LYVR  ESHRR  SYVR  YDLFHMILHSVWYNR |  |
| Probable microtubule-binding protein TANGLED | 9 | MLLFVCPCREDFR  EFQEQNPDIKSMR  EFQEQNPDIKSMR  SMRDVEK  SMRDVEK  CMAR  CMAR  STRGYMK  FPTTAGG |  |
| Actin-related protein 2/3 complex subunit 3 | 8 | ACGCPLLPLKTHIK  GPAPASDSDK  ADVVDEAITFFRANVFFK  NFHVK  LEGCR  LEGCRTLAVGTK  LLNCAYR  LLNCAYRTNGTPNK |  |
| **Photosynthesis and light harvesting** | | | |
| Ribulose-1,5-bisphosphate carboxylase/oxygenase large subunit, partial (chloroplast) | 30 | AGVKDYR  LTYYTPEYK  TKDTDILAAFR  DTDILAAFR  YKGR  ALRLEDLR  LEDLRIPPAYSK  TFIGPPHGIQVER  YGRPLLGCTIKPK  AVYECLR  AVYECLR  GGLDFTKDDENVNSQPFMR  WRDR  DRFLFVAEALFK  FLFVAEALFK  GHYLNATAGTCEEMLKR  AVFAR  DNGLLLHIHR  AMHAVIDR  AMHAVIDR  AMHAVIDRQR  NHGMHFR  MSGGDHIHAGTVVGK  MSGGDHIHAGTVVGK  EVTLGFVDLLR  EVTLGFVDLLRDDYIEK  VALEACVQAR  NEGRDLAR  EGNEIIR  EASKWSP |  |
| Ribulose bisphosphate carboxylase/oxygenase activase 1, chloroplastic isoform X4 | 16 | AGNFRAK  VVCQVDESSQSK  LIRQR  QRYR  VPIIVTGNDFSTLYAPLIR  FYWAPTR  FYWAPTREDR  LVDTFPGQSIDFFGALR  ARVYDDEVR  VYDDEVRK  LLEYGNMLVK  GSSLGDANEDEREK  TPATSSLK  SDNGSCIYDFDEK  VESDATKK  KSGIPEK |  |
| **Plant growth and development** | | | |
| Glycine-rich protein DOT1-like | 7 | AGLNR  GDGGGGDGGK  GDGGGGDGGKGDGDDNGSK  GDGDDNGSK  GDSNGGK  ASDIMLLV  ASDIMLLV |  |
| EPIDERMAL PATTERNING FACTOR-like protein 2 | 7 | MASFTK  YSSQELR  GTCGGVKVGSRPPR  VGSRPPR  VGSRPPRCSVDK  CSVDK  IYHP |  |
| **Detoxification** | | | |
| Rhodanese-like domain-containing protein 19, mitochondrial isoform X2 | 5 | MEVQK  LEEDFEK  NPRFIEQVSSLFGK  DEVFIVGCR  STNEEA |  |
| **Protein with not defined function** | | | |
| Putative cysteine-rich repeat secretory protein 21 | 9 | LVSVPILAVVTIQILFIR  SGFSLNQTNAYLHHK  SPYEENLNRVVR  VVRSISTGNLR  SISTGNLRSGFAHVSNGDTPNTVFVK  GDSYWSK  GGIIWYDNCLLEISSIDTLGK  EKATSNETNAGR  DYTVYAAGDNKLGPMK |  |
| **Hypothetical and uncharacterized proteins** | | | |
| Predicted protein | 11 | EKFDK  VRCNDDDIMQIFSK  FGITR  FENGWPK  VQPPK  SDLTNVK  QAIELCK  SRPCER  NKFNK  FNKHYGK  CYELGSLVQDRMQK |  |
| Hypothetical protein OsI_25859 | 9 | MATPAAAAAAK  ATPAAAAAAK  VQLMK  AHEVAIGELNNLPPSR  AVYQK  SAVTSEQR  HAEGKASR  NHGPYDHVK  NHGPYDHVK |  |
| Uncharacterized protein | 9 | EEYK  IESTHFK  TLLCK  KMEFLSR  RSEISGR  GLFAAK  KEMSR  KEMSR  WMVRGK |  |
| Hypothetical protein CRG98_037683 | 5 | KAMSVIR  EKTTK  GILQVLADMYEKPSTANK  LFNLK  SGTVTAISASTGK |  |
| Hypothetical protein CRG98_010592 | 5 | WLQSDK  IVSCK  IVSCKDR  IEDGWGK  RESDK |  |
| Uncharacterized protein LOC109820622 | 9 | MMQIEDYLYQK  MMQIEDYLYQK  DLFLPLQREAGKPDK  VLDCK  VLDCKALVIIR  ALVIIR  MKLDTMIGAILDER  MMQIEDYLYQK  ISSQSLRS |  |
| Hypothetical protein JCGZ_04055 | 15 | TTPSYVAFTDTER  TTPSYVAFTDTERLIGDAAK  NQVAMNPENTVFDAKR  LIGRR  VIPGPGDKPMIVVR  QFSPEEISSMVLIK  KEIAEAFLGHSIK  NAVVTVPAYFNDSQR  LVHHFVSEFRR  CMETVEKCILDAK  IPKVQQMLSDFFNGK  ITITNDK  ITITNDKGR  IERAIDEALEWLDR  NQLAEVDELEDKLK |  |
| Conserved hypothetical protein | 6 | MWPSSSPRSNGSTR  WPSSSPR  SNGSTR  SNGSTRSR  GAHFGQATRPGFFSK  ACDE |  |
| Hypothetical protein KK1_041423 | 5 | MAMMSFLFNCFVSSSSSPLAK  AMMSFLFNCFVSSSSSPLAK  VSDSSQLNLK  VSDSSQLNLKSTSSEKPTK  SKGAPLVVSYFPVNYYPSR |  |
| Hypothetical protein AXG93_702s1060 | 11 | ESFTHR  SHLKDSR  CYSCQEAAGR  FVYKK  KISAALQPYLPK  EFFHFGLFYFVFIR  IKLGVGMIAK  LGVGMIAK  VASYLKANPDVDTSK  SVHEGGVQIAMTCIVRPTGGSEFYR SVHEGGVQIAMTCIVRPTGGSEFYR |  |
| Hypothetical protein EUGRSUZ_F03718 | 12 | QVLK  GQKIPLFSAAGLPHNEIAAQICR  IPLFSAAGLPHNEIAAQICR  VTLFLNLANDPTIER  IITPR  EEVPGRR  GYPGYMYTDLATIYER  AGRIEGR  QIYPPINVLPSLSR  NIYQSLDLAWTLLR  IFPR  ELLHR |  |
| Predicted protein, partial | 7 | QGLYLARLLNSVMK  LLNSVMK  AGGGHANSHVKVDLGPK  QSKGSK  GISIAGFANWFIWR  LSVKCVR |  |
| Hypothetical protein COLO4_01805, partial | 7 | VQPFDTADPGKQQR  QQRDDAPADHDAR  QPAPR  AELMQR  TEAEVLR  GNGGAIEEVDKEHQR  LIHNAPPKR |  |
| Hypothetical protein F511_11743 | 9 | MDFK  DFKEK  EKSSR  QLKHNFK  HNFKTEENSYPK  AYTNRGTLGQDFTER  VERTGSR  VSSNPR  TTAGHGGNR |  |
| Hypothetical protein GQ55_7G208200 | 12 | MNSDQILEVPDTPDR  MQQSTCPVSSSVVRR  GVAGADICQGSSSGEVK  HVGQK  QRYAMR  YAMR  TLIPQSAYHANSSNCSEIGLSGRNK  LPISLMGKLTCSSER  MGTKTIMIGR  TIQAQLESDELLAR  ASITSGLR  YPGAAR |  |
| Hypothetical protein LR48_Vigan03g182600 | 6 | IYSRK  EMLINVNKFVDNR  FVDNR  DHTALPEEITIRDK  DKEVVCK  EVVCK |  |
| Uncharacterized protein LOC21398768 | 9 | EEYK  IESTHFK  TLLCK  RSEISGR  GLFAAK  RLHVGDYVLVHASR  KEMSR  KEMSR  WMVRGK |  |
| Hypothetical protein GOBAR_AA34479 | 5 | KQSPFK  QSPFK  LQVEELDEWWTHKPK  FGTFK  LLEPP |  |
| Uncharacterized protein LOC111829959 | 5 | FAELTQR  IETLNSK  IETLNSKIK  IKYMEGK  AVQNPK |  |
| Hypothetical protein PHYPA_023536, partial | 5 | AKHCDLWQYHYSGTAYLDFWK  ESSRR  RGDACK  GDACK  FVHRVF |  |
| Hypothetical protein MNEG_5839 | 10 | GTAKQSQPGGLGGLYSIMQTMESK  RQSR  TAESEAEAESQMR  SPRGK  QDEEGVEGEGSPSKK  AVGGARR  ARHGAAAGAADGGGAGQAR  HGAAAGAADGGGAGQARSR  AGAGSKASK  AAAAGPGR |  |
| Unnamed protein product | 7 | LQSLK  TMDDVRSLVSMR  LDQMK  NPVSK  LQTIGSSLTSCTELK  EKLVSTK  DVRNIIDDGEAPFVELFTTDVAENAIGR |  |
| Hypothetical protein VITISV_025075 | 6 | MTNGK  FKSVK  SVKHR  HRVVTESR  LADNR  LGLFEK |  |
| Hypothetical protein TRIUR3_34013 | 6 | ASAVVCK  SGAPVAFR  LLSLMEDVASQTGGLSSTAGAGASQLGR  YNRR  MDKIK  MDKIK |  |
| Hypothetical protein AT4G16060 | 7 | MIALSLSVSLTSLPFSFAFSSTPCK  ASPPR  LLSSDNR  LQGFRR  YSDEEFFQIRCK  FKIDK  LYLR |  |
| Uncharacterized protein A4U43_C01F20550 | 10 | MQPSQLAEEQRK.  AYPLPLLPLQDK  RVMTK  VMTKK  NNGWSQEIEEEMR  EMEEEIEKNLGER  NLGER  SLSELK  TDEDIQEFAGK |  |
| Uncharacterized protein LOC105781050 isoform X2 | 8 | KWMPWK  KWFK  EMKAK  GAGTLK  YMVR  LRLLK  LLKSK  KECIVLDQHAELYR |  |
| Uncharacterized protein LOC108225632 | 9 | SIGSLGIPGK  SSNSR  SKLYGFK  MPQTTIQYIDKQR  TNGPR  SQFLR  TSVTSK  HFSTASQVAICSDSK  EGLNR |  |
| Uncharacterized protein LOC109794716 | 8 | MGNSLR  MGNSLRCCLACVLPCGALDLIR  GNSLR  GSIYFLIPASSLPEK  RHVPR  DNGTFLASKEK  CSWR  CSWRDR |  |
| Hypothetical protein TRIUR3_04918 | 10 | IMDR  VMSPELMDQIRNR  IPISEAIHANGFIGAK  MIRAK  GRLSR  LITILNR  LITILNR  VMTTKQR  LMNENEK  GILPHK |  |
| Hypothetical protein VOLCADRAFT_120394 | 15 | MEHTLR  STVAELETNLATAR  STVAELETNLATARAEVANGK  AARQQTETELR  AEREADMR  IDDLNSQLADADARADAAAAQLMSATNR  IDDLNSQLADADARADAAAAQLMSATNR  ADAAAAQLMSATNR  EAELQQELRQMQMMLDR  QMQMMLDRSQFER  DIRQTISSSEK  QTISSSEKSAK  EQEAMLMAK  GDKPPVPDQQETLPPKLEPMPIPSR  RVSHLDPLDK |  |
| Predicted protein | 9 | MASIARAVAAATASSAALVVTVHSVR  VSTPPIRPAR  LIDEYKGLTK  RAEGR  DVQSVVFGVLQER  EREEAEAER  EEAEAER  EEAEAEREAFK  KIAEVAHDEGLL |  |
| Uncharacterized protein LOC105782132 isoform X1 | 7 | TLASVVGSEEEARK  IYSVCTTRYTGFR  LGELPR  IYADGGRLTR  YTGFCAVMSK  VDRVSEAENDAYLETK  LLDLVMHQILNDIYTRK |  |
| Uncharacterized protein LOC109163844 | 10 | DVTDWNLVAKTLTGGQTTSWPTGDK  DYLLSSHLTSK  MKLDLGK  LDLGKWIYYHILTLTHPR  WIYYHILTLTHPREQK  LLSGDHIK  LAEQLKAR  VADLETVHGIIAK  MSLTDTAAAVDPEK  TDSDSPSNA |  |
| Hypothetical protein GLYMA_01G118500 | 6 | SNSDGGAK  SNSDGGAKGPTAEIITTSCK  GPTAEIITTSCK  LTNIK  NLVEFSLGGMTKEDYLTETCPDGSK  AVMCNTNLSLSR |  |
| Hypothetical protein PRUPE_I003600 | 9 | MAAELTSGQDHALTMASCQDELLAK  IQLIDLK  KLISK  FKFGGEVDDMGAR  ALDRLTPSTVQFK  KDWVQMPSFPFSYK  NWFR  NWFRLTLSFTPECGDYEPEESK  HEVAFMHHEKANQTKPK |  |
| Uncharacterized protein LOC18422773 isoform X1 | 15 | IVDFIDVLDEDGGEHVRVTSSISPEFLLGK  SERDTAK  DSVTTHLQGVAK  EGFGVSHVEETER  RYIEESSEMK  FRSGR  NCTSLLVHDAQDDKDR  WSDIKR  TSVDLK  NLLRASYAQLQTK  HASSVSIPASILK  ELSVMYPKDHQHRPLTEQVNNR  NAFMMGR  NAFMMGR  NAFMMGRSGGNVHK |  |
| Uncharacterized protein LOC111912866 | 10 | MEKAADLVTALEK  ATLMAK  EEDSKMTLIDLVEER  MTLIDLVEER  MTLIDLVEERMK  MKSCFVQK  SCFVQK  NKRPK  RPKRPLSPTSLAAGEK  AGVDYDPLLMK |  |
| Uncharacterized protein LOC111315162 | 25 | MDFHGMKR  MDFHGMKR  VEDCQVR  QPTRNAR  DIASMVKSEVK  QQMQEPSLEEEK  TSVVELHAITEKPQR  ASFAPCNPTAK  NADGCSNKK  ENNLLGDDR  AEVIATEFSKR  VDAANAAGRGMSDQER  IHDDTSEGEMK  VVSPEIASLANVFSPASQVDLAGK  HEFR  LCSAGLTVCIAR  LCSAGLTVCIARSEICNQDEETSLVEDDK  SGNVNYESDKIEVEDDK  KAEELSTDNSSGK  VETAVSHAGYGMCGQQK  STVGIIMGNVLK  DGCQIASGGFSGVTDGSLVER  SELNLLSSQK  SKPSVIQR  RPLEPVWKY |  |
| Hypothetical protein GLYMA_01G118500 | 5 | SNSDGGAKGPTAEIITTSCK  GPTAEIITTSCK  LTNIK  NLVEFSLGGMTKEDYLTETCPDGSK  AVMCNTNLSLSR |  |
| Hypothetical protein PHAVU_003G146800g | 5 | VAVKK  LLLSATVLSHAWHAANILRGVSMSK  GVSMSK  QISFNDR  QLQMER |  |
| Hypothetical protein GLYMA_01G118500 | 5 | SNSDGGAKGPTAEIITTSCK GPTAEIITTSCK  LTNIK  NLVEFSLGGMTKEDYLTETCPDGSK  AVMCNTNLSLSR |  |
| Hypothetical protein OsJ_21377 | 6 | LRQK  SDIQITK  RNYNR  RPCR  VILHSPQR  RFGTK |  |
