# Supplementary material for: Proteome Analysis of the Gametophytes of a Western Himalayan Fern Diplazium maximum Reveals Their Adaptive Responses to Changes in Their Micro-Environment
Source: Front Plant Sci. 2019 Dec 17;10:1623. doi: 10.3389/fpls.2019.01623 (PMC6928197; doi:10.3389/fpls.2019.01623)
Supplement: Table S4 — List of differentially abundant proteins (DAPs) of D. maximum gametophytes that appeared or disappeared in response to differential osmotic potential created by 1 and 3% sucrose (G1 and G3) as compared to no sucrose (G0). [file Table_4.docx]

**Table S4 List of differentially abundant proteins (DAPs) of *D. maximum* gametophytes that appeared or disappeared in response to differential osmotic potential created by 1 and 3% sucrose (G1 and G3) as compared to no sucrose (G0).**

| **Functional classification** | **G1/G0**  **(Appeared)** | **G1/G0**  **(Disappeared)** | **G3/G0**  **(Appeared)** | **G3/G0**  **(Disappeared)** |
| --- | --- | --- | --- | --- |
| Signalling | Gibberellin 2-beta-dioxygenase 1-like isoform X1 | Probable protein phosphatase 2C 21 | Probable calcium-binding protein CML45 | **-** |
|  | Shaggy-related protein kinase kappa isoform X3 |  |  |  |
|  | CRIB domain-containing protein RIC11-like |  |  |  |
|  | Ras-related protein RABB1c-like |  |  |  |
| Stress and Defence | Protein RESTRICTED TEV MOVEMENT 2 | Glutathione S-transferase T3-like | Protein RESTRICTED TEV MOVEMENT 2 | - |
|  | Peroxidase 27-like |  | Peroxidase 27-like |  |
|  |  |  | Phosphopantetheine adenylyltransferase isoform X1 |  |
|  |  |  | 23.5 kDa heat-shock protein |  |
|  |  |  | AP2/ERF domain-containing transcription factor, partial |  |
| Transport and trafficking | AP-4 complex subunit epsilon | - | Exocyst complex component SEC6 isoform X8 | Calmodulin-binding family protein |
|  | Exocyst complex component SEC6 isoform X8 |  |  |  |
|  | Putative protein transport Sec1b |  |  |  |
|  | Mechanosensitive ion channel protein 6-like |  |  |  |
|  | Ras-related protein Rab-2-B |  | Ras-related protein Rab-2-B |  |
| Protein synthesis, folding and turnover | Protein RMD5 homolog A | - | E3 ubiquitin protein ligase DRIP2 | - |
|  | Protein RNA-directed DNA methylation 3-like |  |  |  |
| Metabolism and synthesis of metabolites | Flavanone 3-hydroxylase | Aldehyde dehydrogenase family 2 member C4 | Flavanone 3-hydroxylase | - |
|  |  | Putative pectate lyase 2 | Galacturonate, partial |  |
|  |  |  | 4-hydroxy-3-methylbut-2-enyl diphosphate reductase-like isoform X1 |  |
| Energy metabolism | ATP synthase beta subunit, partial (chloroplast) | - | Cytochrome P450 | ATPase family AAA domain-containing protein 3C |
| Cell wall and cell structure | - | Katanin p80 WD40 repeat-containing subunit B1 homolog isoform X2 | - | - |
|  |  | Probable glycosyltransferase At5g03795 |  |  |
| Plant growth and development | - | - | EPIDERMAL PATTERNING FACTOR-like protein 2 | - |
| Protein with not defined function | - | - | Putative cysteine-rich repeat secretory protein 21 | - |
| Hypothetical and uncharacterized proteins | Uncharacterized protein LOC109794716 | Predicted protein | Uncharacterized protein LOC105782132 isoform X1 | Hypothetical protein OsI_25859 |
|  | Hypothetical protein TRIUR3_04918 | Hypothetical protein CRG98_010592 | Uncharacterized protein LOC109163844 | Unnamed protein product |
|  | Hypothetical protein VOLCADRAFT_120394 | Hypothetical protein AXG93_702s1060 | Uncharacterized protein LOC111912866 | Hypothetical protein AT4G16060 |
|  | Predicted protein | Predicted protein, partial | Uncharacterized protein LOC111315162 | Uncharacterized protein LOC105781050 isoform X2 |
|  | Uncharacterized protein LOC105782132 isoform X1 | Hypothetical protein GQ55_7G208200 | Hypothetical protein GLYMA_01G118500 |  |
|  | Uncharacterized protein LOC109163844 | Hypothetical protein PHYPA_023536, partial | Hypothetical protein PHAVU_003G146800g |  |
|  | Hypothetical protein GLYMA_01G118500 | Hypothetical protein TRIUR3_34013 | Hypothetical protein GLYMA_01G118500 |  |
|  | Hypothetical protein PRUPE_I003600 |  | Hypothetical protein OsJ_21377 |  |
|  | Uncharacterized protein LOC18422773 isoform X1 |  |  |  |
