# Supplementary figures and images for: Proteome Analysis of the Gametophytes of a Western Himalayan Fern Diplazium maximum Reveals Their Adaptive Responses to Changes in Their Micro-Environment
Source: Front Plant Sci. 2019 Dec 17;10:1623. doi: 10.3389/fpls.2019.01623 (PMC6928197; doi:10.3389/fpls.2019.01623)

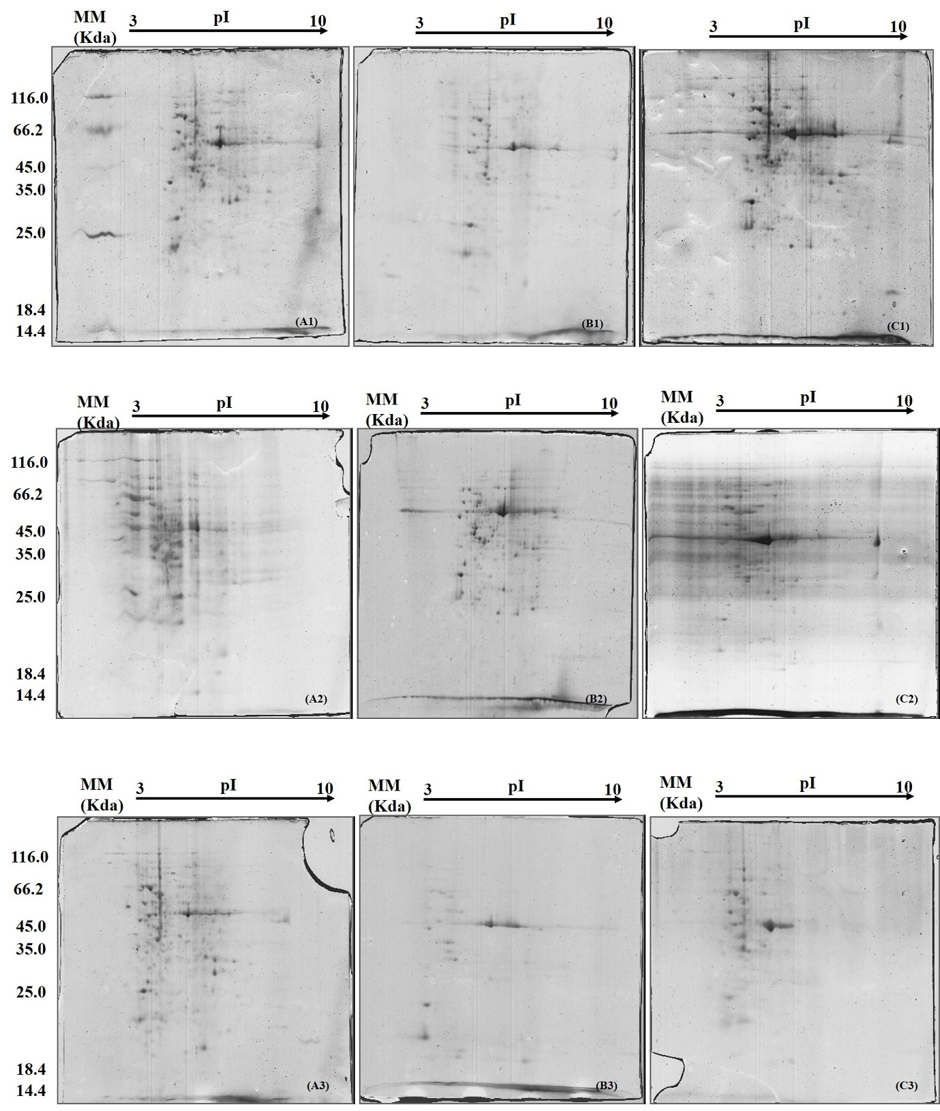

Supplement: Figure S1 — Three biological replicates of 2-DE gels from the gametophytes of D. maximum. The gels were stained with Coomassie Brilliant Blue G-250. Molecular weight (MM) in kDa and pI of proteins are indicated on the left and top of the gels, respectively. (A1, B1, C1) G0 (A2, B2, C2) G1 (A3, B3, C3) G3. [file Image_1.tif]
